# Human coronavirus HKU1 recognition of the TMPRSS2 host receptor

**DOI:** 10.1016/j.cell.2024.06.006

**Published:** 2024-07-03

**Authors:** Matthew McCallum, Young-Jun Park, Cameron Stewart, Kaitlin R. Sprouse, Amin Addetia, Jack Brown, M. Alejandra Tortorici, Cecily Gibson, Emily Wong, Margareta Ieven, Amalio Telenti, David Veesler

**Affiliations:** 1 Department of Biochemistry, University of Washington, Seattle, Washington, USA; 2 Howard Hughes Medical Institute, Seattle, WA 98195, USA; 3 Vir Biotechnology, San Francisco, CA 94158, USA; 4 Laboratory of Clinical Microbiology, Vaccine & Infectious Disease Institute, University of Antwerp, Antwerp, Belgium

**Keywords:** Coronaviruses, HKU1, spike glycoprotein, TMPRSS2, species tropism, neutralizing antibodies, glycan shielding, immune evasion

## Abstract

The human coronavirus HKU1 spike (S) glycoprotein engages host cell surface sialoglycans and transmembrane protease serine 2 (TMPRSS2) to initiate infection. The molecular basis of HKU1 binding to TMPRSS2 and determinants of host receptor tropism remain elusive. We designed an active human TMPRSS2 construct enabling high-yield recombinant production in human cells of this key therapeutic target. We determined a cryo-electron microscopy structure of the HKU1 RBD bound to human TMPRSS2 providing a blueprint of the interactions supporting viral entry and explaining the specificity for TMPRSS2 among human type 2 transmembrane serine proteases. We identified TMPRSS2 orthologs from five mammalian orders promoting HKU1 S-mediated entry into cells along with key residues governing host receptor usage. Our data show that the TMPRSS2 binding-motif is a site of vulnerability to neutralizing antibodies and suggest that HKU1 uses S conformational masking and glycan shielding to balance immune evasion and receptor engagement.

## Graphical Abstract

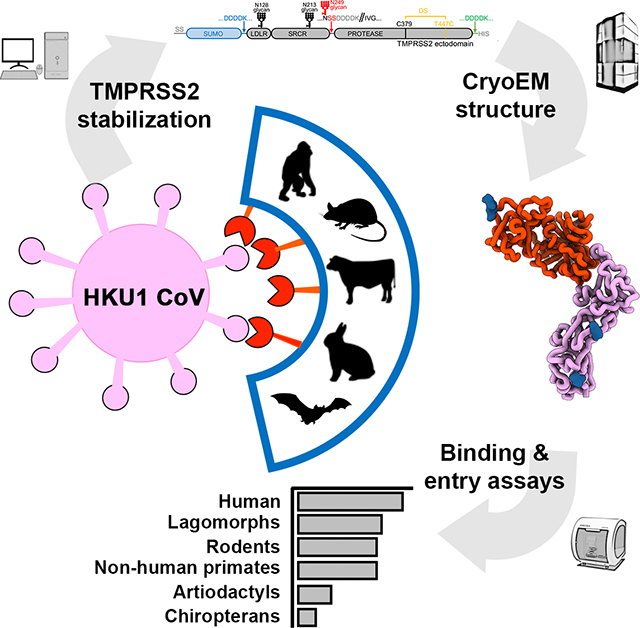

## INTRODUCTION

HKU1 is one of the four β-coronaviruses known to circulate in humans seasonally. It was initially discovered in 2005 in nasopharyngeal aspirates obtained from a 71-year-old man with pneumonia in Hong Kong^1^. Retrospective investigations identified HKU1 in nasopharyngeal aspirates collected between 1995 and 2002 from symptomatic individuals on several continents^1–4^ and more HKU1 strains were sequenced and isolated thereafter^5,6^. Although HKU1 is largely considered to be a ‘common cold’ coronavirus, causing mild respiratory infections, it also causes severe illness, particularly (but not only) for children, the elderly and immunocompromised individuals^7–9^. Furthermore, its seroprevalence in multiple independent studies indicates that HKU1 continuously circulates in the human population and is therefore endemic^8^.

Coronavirus infections are initiated by interaction of the trimeric spike (S) glycoprotein with a host cell receptor leading to fusion of the viral and host membrane and genome delivery^10^. To date, only a finite number of proteinaceous receptors have been identified as promoting coronavirus infection and virtually all of them are host transmembrane proteases: angiotensin-converting enzyme 2 (ACE2) for severe acute respiratory syndrome coronavirus (SARS-CoV-1)^11^, SARS-CoV-2^12–14^, NL63^15,16^ and several bat-borne merbecoviruses^17–19^; aminopeptidase N (APN) for 229E^20^, CcoV-HuPn-2018^21^, PDCoV^22^, TGEV/PRCV^23^ and PEDV^24^; dipeptidyl-peptidase 4 (DPP4) for MERS-CoV^25^ and HKU4-related merbecoviruses^26–28^; and carcinoembryonic antigen-related cell adhesion molecule 1a for MHV A59^29^. TMEM106B has additionally been described as a receptor mediating ACE2-independent SARS-CoV-2 cell entry^30^.

Embecoviruses, such as HKU1 and OC43, are set apart from other coronaviruses as they harbor both S trimers and hemagglutinin-esterase (HE) dimers at their surface. Embecovirus S recognize 9-O-acetylated sialosides^31–34^, linked to oligosaccharides at the host cell surface, whereas HE is a receptor-destroying enzyme^35,36^, with sialate-O-acetyl-esterase activity, facilitating release of viral progeny from infected cells and escape from attachment to non-permissive host cells or decoys. Furthermore, a recent study showed that transmembrane protease serine 2 (TMPRSS2) is an entry receptor for HKU1^37^, which is likely engaged upon S conformational changes triggered by α2,8-linked 9-O-acetylated disialosides binding^38^. The molecular basis of HKU1 recognition of the human TMPRSS2 receptor along with the determinants of host receptor tropism, however, remain elusive.

TMPRSS2 is part of the type 2 transmembrane serine protease family that encompasses several cell surface-expressed trypsin-like serine proteases participating in epithelial homeostasis and TMPRSS2 genomic rearrangements are common in prostate cancer^39^. Furthermore, TMPRSS2 proteolytically activates the S glycoprotein of many coronaviruses, including SARS-CoV-2^40,41^, SARS-CoV-1^42,43^, MERS-CoV^44,45^, 229E^46^, as well as influenza virus hemagglutinins^47,48^. As a result, TMPRSS2 inhibitors have been proposed as candidates for preventing or treating these viral infections^49–51^. Recombinant production of TMPRSS2 and related type 2 transmembrane serine proteases has proven exceptionally challenging, hindering studies of these important therapeutic targets.

Here, we designed a human TMPRSS2 glycoprotein construct enabling high-yield recombinant production of the active mature enzyme ectodomain in human cells, which will expedite biochemical studies and drug screening globally. We determined a cryo-electron microscopy (cryoEM) structure of the human TMPRSS2 ectodomain bound to the HKU1 S receptor-binding domain (RBD), providing a blueprint of the interactions mediating receptor engagement, specificity and host receptor tropism. We reveal that the TMPRSS2-binding motif is a key site of vulnerability for HKU1 that becomes exposed upon sialoglycan-mediated RBD opening. Our findings elucidate how this human coronavirus gains access to target host cells and balances receptor recognition and immune evasion, informing vaccine design.

## RESULTS

### Design of an active human TMPRSS2 construct

Given the therapeutic relevance of human TMPRSS2, we sought to design a strategy enabling recombinant production of native enzyme from human cells. Although a recent study reported producing the active TMPRSS2 ectodomain (dasTMPRSS2) using *Spodoptera frugiperda* Sf9 cells^52^, insect cell expression is inherently time-consuming and does not faithfully recapitulate the chemical composition of protein glycosylations^53,54^. Furthermore, the replacement of the native TMPRSS2 autoactivation cleavage site with an enterokinase cleavage sequence in dasTMPRSS2 removes the endogeneous N249 glycosylation motif and requires an additional proteolytic activation step, adding to the laborious nature of this purification.

**Figure 1.**
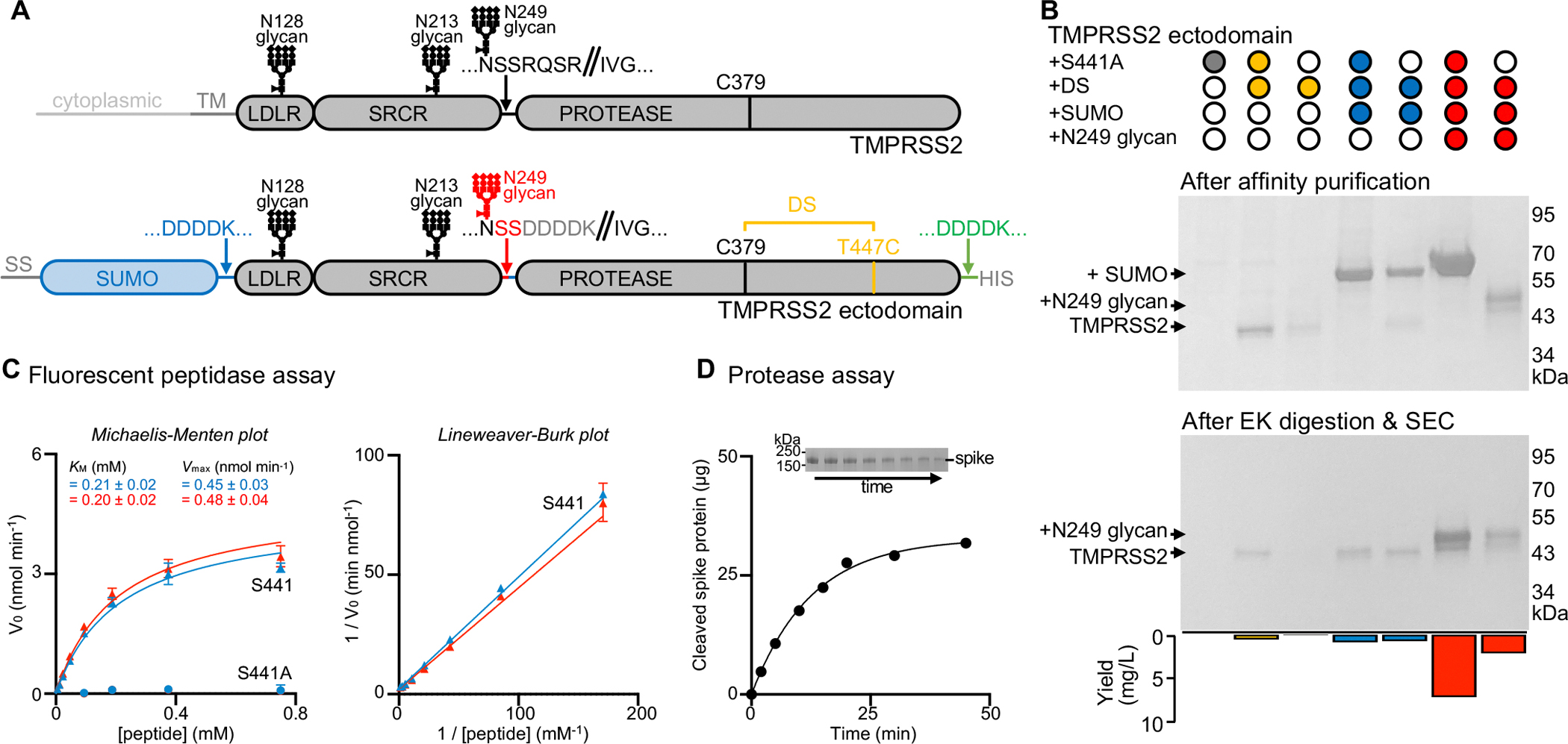
Design and functional characterization of an enzymatically active human TMPRSS2 construct (A) Schematic representation of the domain architecture of human TMPRSS2 and of the ectodomain construct designed for mammalian expression. The sequences of the constructs tested can be found in Table S1. Designed features are a C379-T447C disulfide bond (DS, yellow), an enterokinase-cleavable N-terminal SUMO fusion (blue), and reintroduction of the N249-linked glycosylation motif (red) relative to a previously described insect cell expression construct (dasTMPRSS2)^52^. An enterokinase-cleavable C-terminal octa-histidine tag is also present (green). TM: transmembrane domain. LDLR: low-density lipoprotein receptor domain; SRCR: scavenger receptor cysteine-rich domain; PROTEASE: trypsin-like serine protease domain. SS: signal sequence. DDDDK: enterokinase (EK) cleavage sequence. //: scissile bond leading to TMPRSS2 zymogen activation. (B) Non-reducing sodium dodecyl-sulfate polyacrylamide gel electrophoresis (SDS-PAGE) of purified TMPRSS2 constructs with or without S441A, the C379-T447C disulfide bond (DS), an N-terminal SUMO fusion, and the native glycan at position N249 (as indicated above the gels). Proteins were expressed and affinity purified in parallel, and then normalized to a standard volume to enable comparisons of relative yields and purity after affinity purification (top) and after EK digestion and SEC purification (middle). The final yield of purified TMPRSS2 per liter of Expi293 expression medium is indicated as a bar graph (bottom). (C) Michaelis–Menten plot (left) and Lineweaver-Burke plot (right) of initial reaction velocities with various concentrations of fluorescent Boc-QAR-AMC peptide substrate, at 22 °C, in the presence of 6.8 nM of the TMPRSS2 ectodomain harboring either the DS and SUMO (blue triangles) or the DS, SUMO, and N249 glycan (red triangles) modifications. S441A TMPRSS2 ectodomains harboring either the DS and SUMO (blue circles) or the DS, SUMO, and N249 glycan (red circles) modifications were used as a negative control. *K*_*M*_ and *V*_*max*_ were determined through fitting using GraphPad Prism. Data are shown as the geometric mean and standard deviation of three technical replicates. (D) Proteolytic processing of 40 μg of SARS-CoV-2 S 2P (S2P) by 0.5 μM human TMPRSS2 ectodomain harboring the DS and SUMO modifications over 45 min at 37°C, analyzed by reducing SDS-PAGE (inset) and quantified by densitometry. The inset on top shows the decreasing intensity of the S2P band over time used for these calculations. The full gel can be seen in Fig. S1D. See also Figure S1 and Table S1.

To overcome these limitations, we designed an ectodomain construct enabling high-yield and rapid recombinant production of active human TMPRSS2. We first observed that we could not detect dasTMPRSS2 expression in Expi293 human cells even after introducing the S441A residue mutation to disable proteolytic activity and potential cytotoxicity during expression (Figure 1A–B, Figure S1 and Table S1). Human TMPRSS2 harbors an unpaired and solvent-exposed cysteine at position 379 (in the protease domain), which otherwise participates in a disulfide bond with the residue equivalent to T447 in most related type 2 transmembrane serine proteases^52^. We therefore introduced the T447C residue substitution to allow formation of a C379-C447 disulfide bond, which was sufficient to detect TMPRSS2 expression, albeit with low yield (Figure 1A–B, Figure S1 and Table S1). Fusion of an enterokinase-cleavable N-terminal small ubiquitin-like modifier (SUMO) tag increased production yield approximately 2-fold, relative to the construct lacking the SUMO fusion (Figure 1A–B, Figure S1 and Table S1). Finally, reintroduction of two native serine residues, corresponding to positions 250 and 251 of the human TMPRSS2 sequence, restored the N-linked glycan at position N249 and improved production yield 8-fold compared to the glycan knockout construct (Figure 1A–B, Figure S1 and Table S1).

Reintroduction of the S441 catalytic residue reduced yields ~3 fold relative to S441A-harboring TMPRSS2 (Figure 1A–B, Figure S1 and Table S1). We noted that the TMPRSS2 construct harboring the wildtype S441 residue, T447C, the N-terminal SUMO fusion, and the native N249 glycan autocatalytically processed the enterokinase cleavage sequences during expression, leading to zymogen activation and SUMO cleavage/removal (Figure 1A–B, Figure S1 and Table S1). Consistent with this interpretation, the catalytically inactive S441A-harboring TMPRSS2 mutant remains uncleaved. Overall, our designed constructs streamline TMPRSS2 production yielding 7 mg of purified S441A TMPRSS2 or 2 mg of self-activating S441 TMPRSS2 per liter of human Expi293 cells, 4 days after transfection, which will accelerate studies of this key therapeutic target.

Purified TMPRSS2 is highly active in a fluorescent peptidase assay, using the fluorogenic Boc-Gln-Ala-Arg-7-aminomethylcoumarine (Boc-QAR-AMC) peptide substrate, and its activity can be modeled with Michaelis-Menten kinetics (Figure 1C and Figure S1). We determined a *K*_*M*_ of 200 μM and V_max_ of 0.5 nmol/min for Boc-QAR-AMC, similar to dasTMPRSS2 purified from insect cells^52^. Given that reintroduction of the N249 glycan did not significantly alter TMPRSS2 enzyme kinetics, the observed autocatalytic cleavage likely resulted from a high TMPRSS2 concentration during production rather than enhanced activity (Figure 1C and Figure S1). TMPRSS2 is also active using the SARS-CoV-2 S ectodomain trimer as a substrate (Figure 1D).

### Structure of the TMPRSS2-bound HKU1 RBD

**Figure 2.**
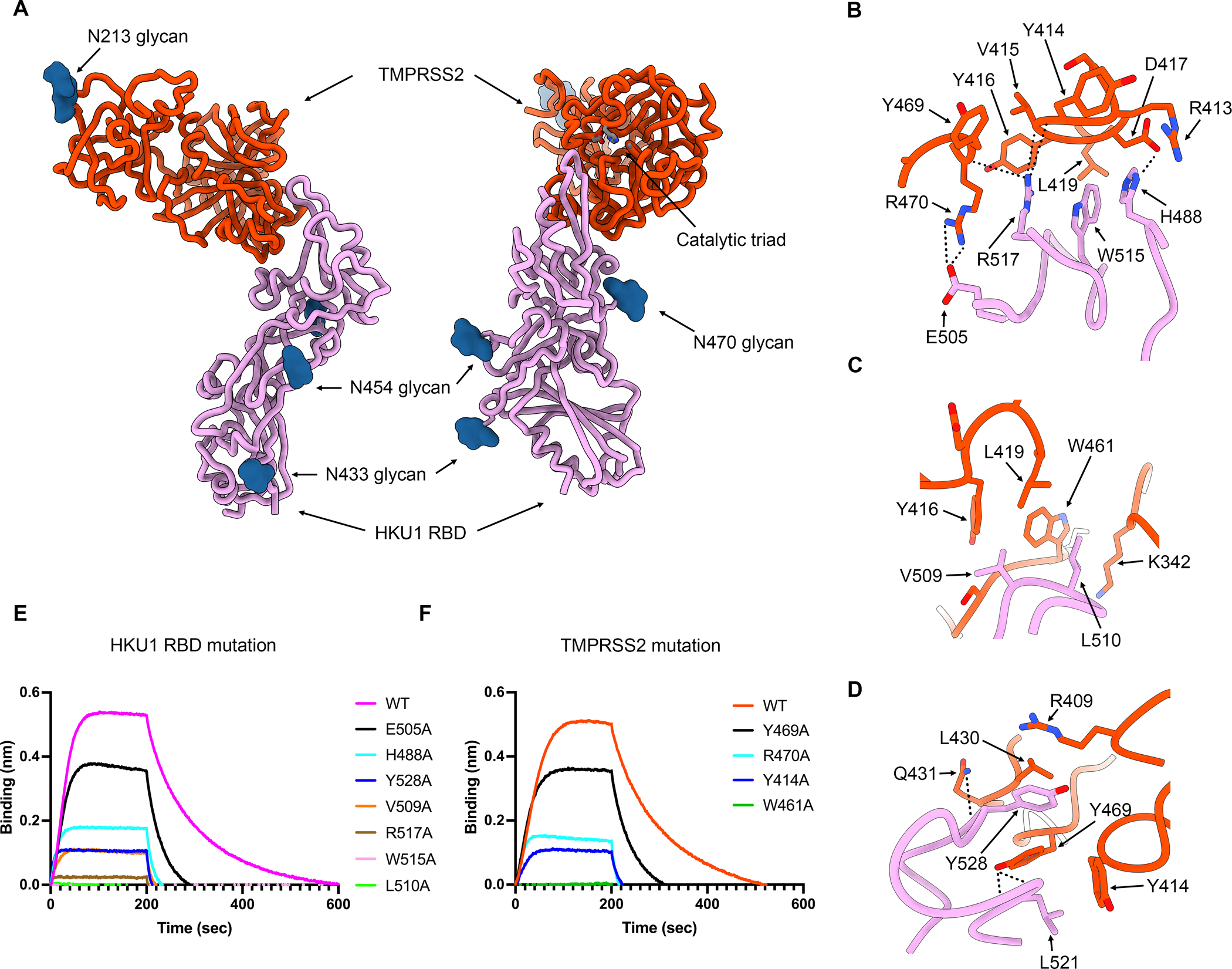
Molecular basis of human TMPRSS2 recognition by the HKU1 RBD. (A) Ribbon diagrams in two orthogonal orientations of the cryoEM structure of the HKU1 RBD (pink) bound to the human TMPRSS2 ectodomain (orange) at 2.9Å resolution. This TMPRSS2 construct harbors the S441A catalytic inactivating substitution and the DS stabilizing mutation and was activated by enterokinase cleavage. The TMPRSS2 catalytic triad residues (H296, D345 and S441A) are shown as sticks colored by heteroatom and N-linked glycans are rendered as blue spheres. (B-D) Zoomed-in views of the interface highlighting key interactions between the HKU1 RBD and the human TMPRSS2 peptidase domain. Select polar interactions are shown as black dotted lines. (E) Binding of S441A TMPRSS2 (same construct as Figure 2A–D) to the wildtype (WT) isolate N1 and to the H488A, E505A, V509A, L510A, W515A, R517A and Y528A HKU1 RBD interface mutants immobilized on biolayer interferometry streptavidin (SA) biosensors. (F) Binding of the wildtype (WT) and the Y414A, W461A, Y469A and R470A human TMPRSS2 (same construct as Figure 2A–D with S441 instead of S441A) interface mutants to the wildtype isolate N1 HKU1 RBD immobilized on biolayer interferometry SA biosensors. See also Figures S2–S3 and Table 1.

To understand the molecular basis of HKU1 engagement of its host receptor, we characterized an HKU1 isolate N1 (genotype A) RBD (which corresponds to the first genome sequence characterized^1^) in complex with the cleaved human TMPRSS2 ectodomain harboring the S441A substitution of the catalytic nucleophile. We used cryoEM to determine a structure of this 85kDa complex at 2.9 Å resolution revealing that the TMPRSS2 catalytic domain is engaged by the HKU1 receptor-binding motif (RBM) which is inserted in between two β-strands of the RBD core β-sheet (Figure 2A–D, Figure S2 and Table 1). No major conformational changes occur upon binding besides small scale structural rearrangements of the HKU1 RBM mostly centered on the β-hairpin spanning residues 505–517, as compared to the isolated RBD structure^55^ (Figure S3).

Receptor engagement leads to burial of surfaces of approximately 800 Å^2^ from each of the two binding partners using polar interactions and shape complementarity. The HKU1 RBM comprises residues 488, 505, 507–512, 515, 517–518, 520–522, 527–530 532, and 554 to recognize TMPRSS2 residues 340–342, 409, 412–417, 419, 430–431, 461–463 and 468–470 (Figure 2A). Key interactions include (i) the H488_HKU1_ imidazole side chain forming electrostatic contacts with the D417_TMPRSS2_ side chain carboxylate (Figure 2B); (ii) the E505_HKU1_ side chain carboxylate forming a salt bridge with the R470_TMPRSS2_ side chain guanidinium (Figure 2B); (iii) the V509_HKU1_ side chain establishing van der Waals contacts with the Y416_TMPRSS2_, L419_TMPRSS2_ and W461_TMPRSS2_ side chains (Figure 2C); (iv) the L510_HKU1_ side chain inserting in a surface-exposed cleft and contacting the aliphatic part of the K342_TMPRSS2_ side chain and the W461_TMPRSS2_ indol ring (Figure 2C); (v) the W515_HKU1_ side chain forming van der Waals contacts with Y416_TMPRSS2_, D417_TMPRSS2_ and L419_TMPRSS2_ (Figure 2A); (vi) the R517_HKU1_ side chain electrostatically interacting with the Y414_TMPRSS2_, V415_TMPRSS2_ and Y469_TMPRSS2_ backbone carbonyls (Figure 2A); (vii) the L521_HKU1_ side chain which is sandwiched between the Y414_TMPRSS2_ and Y469_TMPRSS2_ side chains and the L521 backbone amide and carbonyl that are hydrogen-bonded to the Y469_TMPRSS2_ side chain phenol (Figure 2D); and (viii) the Y528_HKU1_ side chain interacting with the R409_TMPRSS2_, L430_TMPRSS2_ and Y469_TMPRSS2_ side chains and the Y528_HKU1_ backbone carbonyl which is hydrogen-bonded to the Q431_TMPRSS2_ amide side chain (Figure 2D). As none of the HKU1 S or TMPRSS2 N-linked glycans contribute to the binding interface, the previously reported HKU1 S pseudovirus sensitivity to neuraminidase treatment of target cells^37^ likely resulted from removal of sialoglycan receptors, thereby dampening entry.

### Validation of the TMPRSS2-bound HKU1 RBD cryoEM structure

To functionally validate the role of key interacting residues identified in our cryoEM structure, we evaluated binding of the cleaved but catalytically inactive S441A TMPRSS2 ectodomain to seven isolate N1 HKU1 RBD point mutants harboring an alanine substitution at the receptor-binding interface. The HKU1 RBD L510A and W515A mutations had the strongest effect among the mutants tested, completely preventing binding (Figure 2E and Figure S4A). R517A had the next strongest effect, severely abrogating recognition, followed by V509A, Y528A, H488A and E505A (Figure 2E and Figure S4A). The deleterious impact of the HKU1 RBD W515A and R517A mutations on TMPRSS2 binding concur with prior biochemical data^37,55^, underscoring the key role of these two viral residues. Furthermore, we found that the TMPRSS2 W461A, Y414A, R470A and Y469A interface mutants had lost or reduced binding to the HKU1 RBD relative to the wildtype TMPRSS2 ectodomain (Figure 2F and Figure S4B). These data demonstrate that the HKU1 S and TMPRSS2 residues identified as main contributors to binding in our cryoEM structure are mediating attachment of the RBD to its host receptor and that mutations of these residues to alanine dampens TMPRSS2 binding.

### HKU1 RBD binding inhibits TMPRSS2 activity

**Figure 3.**
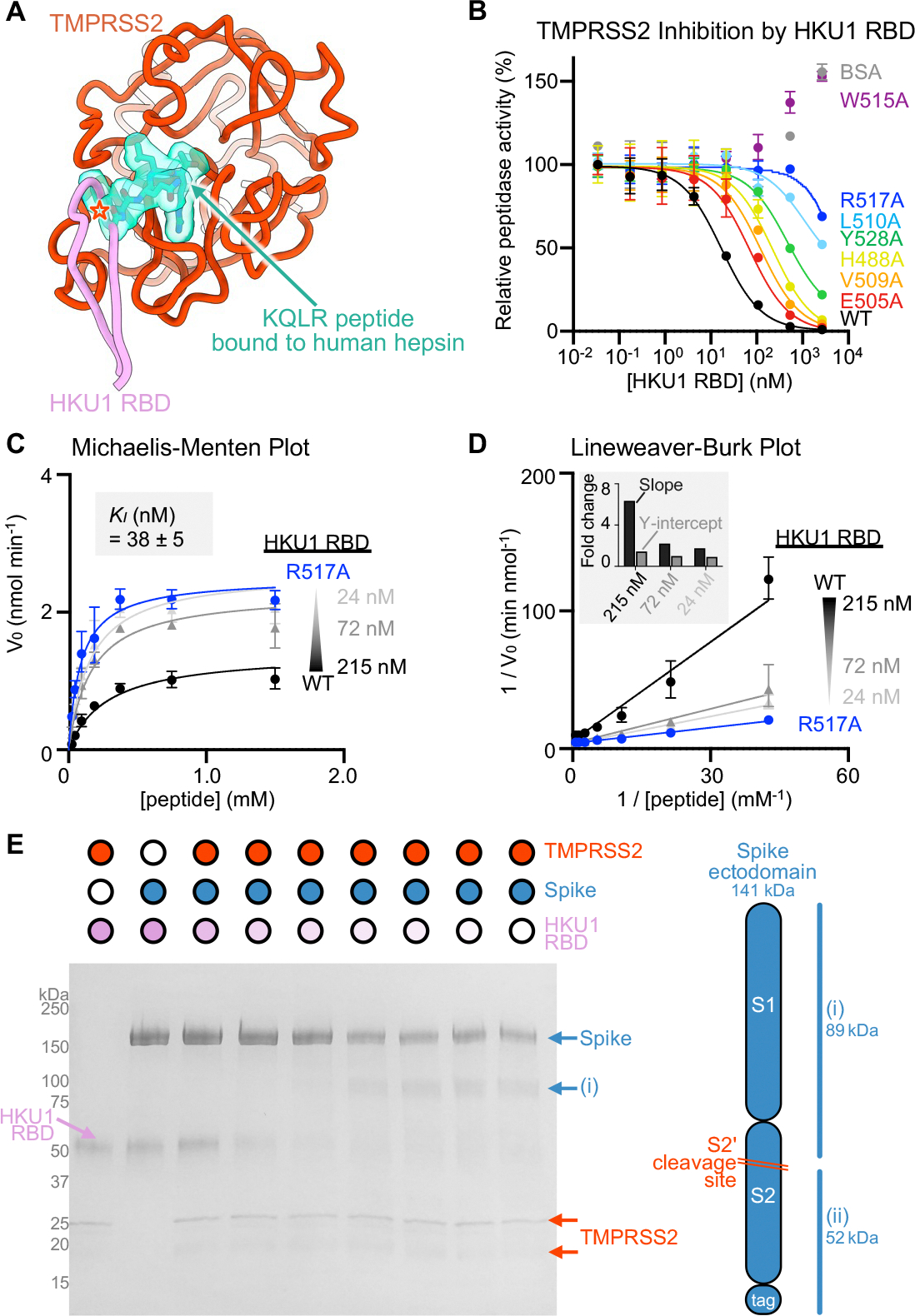
HKU1 RBD binding inhibits human TMPRSS2 activity (A) Superimposition of the KQLR-bound (teal) hepsin structure (PDB 1Z8G^56^) to the HKU1-bound TMPRSS2 (orange) structure showing that the ligand would be precluded sterically from binding to the active site upon attachment of the HKU1 RBD (assuming an identical binding mode of the ligand). Only the TMPRSS2 peptidase domain is shown and hepsin is omitted for clarity. Steric clashes are indicated with an orange star. (B) Assessment of inhibition of human TMPRSS2 activity by the wildtype (WT) isolate N1 and interface mutant HKU1 RBDs using the fluorescent Boc-QAR-AMC peptide substrate in the presence of 3.4 nM of the TMPRSS2 ectodomain harboring the DS, SUMO, and N249 glycan. Data are shown as the geometric mean and standard deviation of 2–3 technical replicates. (C-D) Michaelis–Menten plot (C) and Lineweaver-Burke plot (D) of initial reaction velocities in function of the concentration of fluorescent Boc-QAR-AMC peptide substrate, at 22 °C, in the presence of 5 nM TMPRSS2 and the wildtype (WT) isolate N1 or R517A HKU1 RBDs. The inhibitor constant *K*_*I*_ was determined through fitting competitive inhibition using GraphPad Prism. Data are shown as the geometric mean and standard deviation of three technical replicates. The inset in panel D shows the relative change in slope (*K*_*M*_/*V*_*max*_) and y axis intercept (1/*V*_*max*_) for the different concentrations of wildtype HKU1 RBD relative to the R517A RBD at a concentration of 215 nM. (E) Reducing SDS-PAGE analysis of proteolytic processing at 37°C of 5 μg of SARS-CoV-2 S 2P (S2P) by 0.5 μM human TMPRSS2 ectodomain harboring the DS and SUMO modifications preincubated with various concentrations of the HKU1 isolate N1 RBD. See also Figure S4.

Upon binding, the HKU1 RBM β-hairpin (comprising residues 505–517) is positioned in close proximity to the active site albeit without contacting the catalytic triad residues (H296, D345 and S441) (Figure 2A). Superimposing the HKU1 RBD-bound TMPRSS2 structure with that of the KQLR-bound human hepsin (TMPRSS1)^56^ indicates that the HKU1 RBM would sterically compete with binding of this substrate to the TMPRSS2 active site, assuming an identical binding mode of the ligand (Figure 3A). To assess a possible impact on TMPRSS2 enzymatic activity, we quantified proteolytic processing of the Boc-QAR-AMC peptide substrate in the presence of various concentrations of the HKU1 RBD. TMPRSS2 proteolytic activity was inhibited by the HKU1 RBD in a dose-dependent manner with an inhibition constant K_*I*_ of 38 +/− 5 nM (Figure 3B–D and Figure S5). Furthermore, the seven HKU1 RBD alanine point mutants at the receptor-binding interface had dampened inhibitory effect on TMPRSS2 activity (Figure 3B and Figure S5), concurring with the binding reductions observed by biolayer interferometry (Figure 2E). The W515A HKU1 RBD mildly increased TMPRSS2 peptidase activity, as was the case for bovine serum albumin (BSA), suggesting non-specific TMPRSS2 stabilization. Michaelis-Menten kinetics analysis of TMPRSS2 activity in the presence of various concentrations of the HKU1 RBD suggested a mechanism of action through competitive inhibition (Figure 3C–D), in line with partial occluding of the active site observed structurally. Furthermore, the HKU1 RBD inhibited cleavage of the purified SARS-CoV-2 S 2P trimer in a dose-dependent manner, as evaluated by SDS-PAGE (Figure 2F). These results underscore the lack of requirement for TMPRSS2 enzymatic activity to promote HKU1 entry, as the S441A inactive mutant was shown to support HKU1 S-mediated pseudovirus entry with comparable levels to wildtype TMPRSS2^37^.

### Molecular basis of HKU1 specificity for TMPRSS2

**Figure 4.**
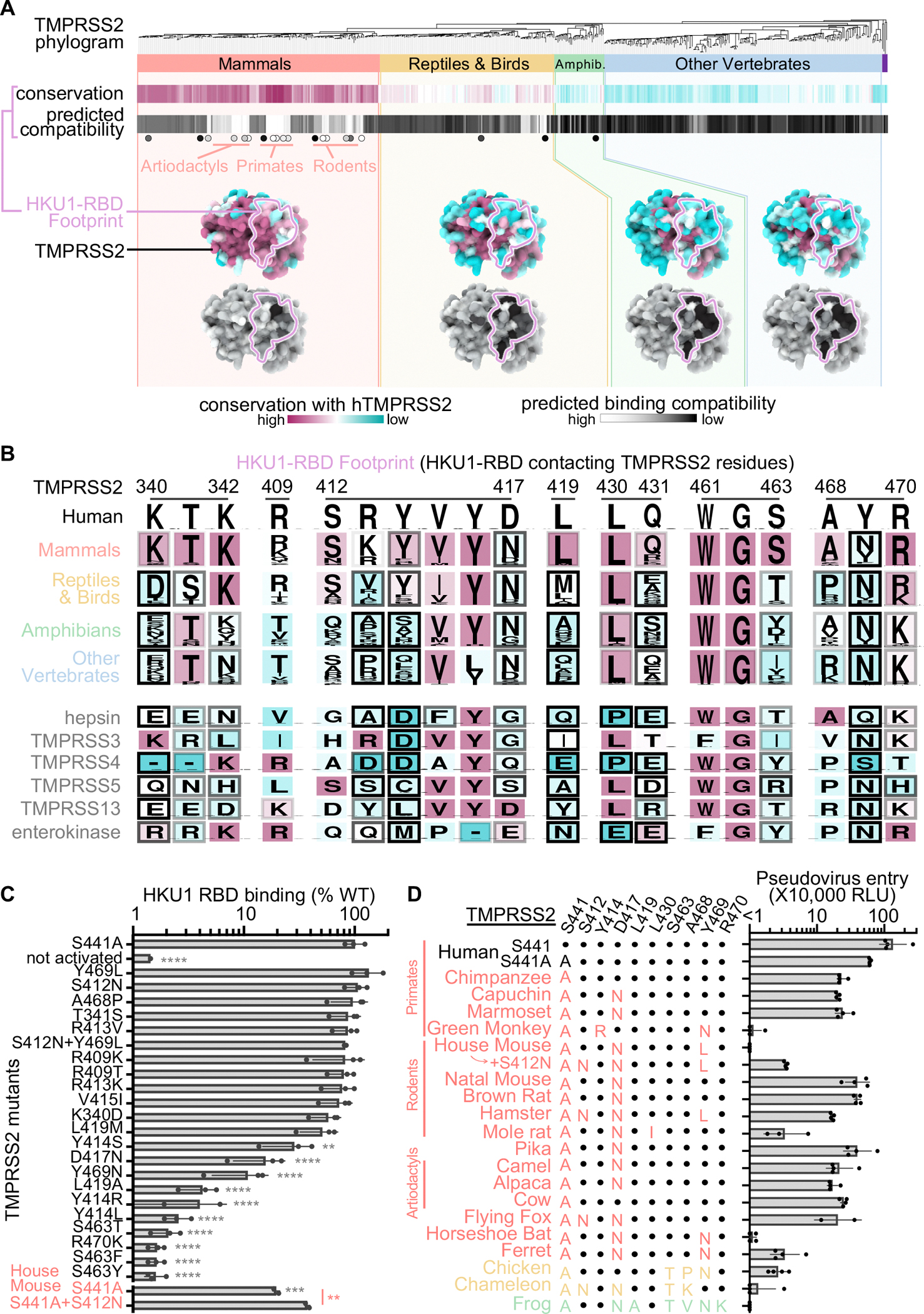
Molecular determinants of HKU1 utilization of human TMPRSS2 and host receptor tropism. (A, top) Neighbor-Joining phylogram of TMPRSS2 orthologs, clustered in red (mammals), yellow (reptiles/birds), green (amphibians), and blue (other vertebrates). The tree is rooted on TMPRSS3 4, 5, and 13 (purple) and was generated with iTOL^92^. (A, middle) The average amino acid sequence conservation (BLOSUM62) relative to human TMPRSS2 of the HKU1 RBD-contacting residues in each TMPRSS2 ortholog is rendered from maroon (conserved) to cyan (not conserved). To calculate the predicted binding compatibility of each TMPRSS2 ortholog, a score for each residue substitution was calculated by subtracting the ProteinMPNN^57^ probability score of apo TMPRSS2 residues from that of the HKU1 RBD-bound TMPRSS2; the average score across HKU1 RBD-contacting is rendered from white (highest score, eg. human TMPRSS2) to black (lowest score). TMPRSS2 orthologs for which we evaluated their ability to support HKU1 S-mediated entry into cells are labeled as circles shaded from white (entry) to black (no entry) proportionally to the magnitude of entry detected in panel (D). (A, bottom) Surface rendering of the human TMPRSS2 peptidase domain colored according to amino acid sequence conservation (maroon to cyan) and ProteinMPNN predicted binding compatibility scores (black to white). (B) Logo plot analysis of sequence conservation with human TMPRSS2 of the amino acid residues interacting with the HKU1 RBD from the TMPRSS2 ortholog clusters shown in panel A (top) and from members of the TMPRSS protease subfamily (bottom) colored from maroon (conserved) to cyan (not conserved). Rendered with Weblogo^93^. The shade of the frame for each residue represents the predicted binding compatibility score from ProteinMPNN. (C) Binding of TMPRSS2 point mutants (corresponding to polymorphisms identified in TMPRSS2 orthologs or in human SNPs) to the HKU1 RBD immobilized on biolayer interferometry SA biosensors. These TMPRSS2 constructs harbored S441 (unless otherwise noted), DS stabilizing mutations, the N249 glycan, and SUMO tag; these constructs auto-activated during purification – except for S441A constructs which were activated by enterokinase – simultaneously removing the SUMO tag. Data are shown as the area under the binding response curve normalized to the signal of wildtype (WT) TMPRSS2. Each data point is a biological replicate which corresponds to the average of 1–3 technical replicates presented in Figure S5 except for uncleaved S441A human TMPRSS2 which shows a single biological replicate with two technical replicates. Bar: mean with standard deviation. Not activated: uncleaved S441A TMPRSS2. Means were compared with cleaved S441A TMPRSS2 by one-way ANOVA (gray asterisks). For the means of the S441A and S441A/S412N House Mouse TMPRSS2 ectodomains, the comparison used an unpaired two-tailed t-test (red asterisk). **: p<0.01, ***: p<0.001, ****: p<0.0001. (D) HKU1 S VSV entry into HEK293T cells transiently transfected with one of the indicated TMPRSS2 orthologs. Key TMPRSS2 amino acid residues interacting with the HKU1 RBD (along with the S441 catalytic serine) are indicated to highlight conserved and divergent positions relative to human TMPRSS2. Data are shown as the mean and standard deviation of 2–4 biological replicates each comprising 2–3 technical replicates. See also Figures S5–S6.

Our structural data provide a blueprint to understand the HKU1 specificity for the TMPRSS2 receptor. Amino acid sequence alignment of TMPRSS2 orthologs reveals that the HKU1-binding site is mostly conserved among mammals, partially conserved in reptiles and birds, and poorly conserved in amphibians and other vertebrates (Figure 4A–B). We scored the compatibility with the HKU1 RBD of residues mutated relative to human TMPRSS2 using proteinMPNN^57^ and observed high compatibility probabilities for mammalian TMPRSS2 residues (especially Primates, Rodents, and Artiodactyls) whereas non-mammalian TMPRSS2 residues scored lower (Figure 4A). Among weakly conserved residues, mutations at positions Y414, D417, and Y469 were predicted to have the greatest reductions on HKU1 RBD binding to mammalian TMPRSS2 orthologs (Figure 4B). Moreover, mutations at positions K340, T341, K342, R413, Y414, D417, L419, Q431, S463, A468, Y469, and R470 were anticipated to particularly dampen HKU1 RBD binding to non-mammalian TMPRSS2s.

To test the effect of some of these mutations, we expressed human TMPRSS2 harboring the K340D, T341S, R409T/K, S412N, R413K/V, Y414S/L/R, V415I, D417N, L419A/M, S463T/Y/F, A468P, Y469N/L, R470K or S412N+Y469L substitutions and analyzed binding to the HKU1 isolate N1 wildtype RBD (Figure 4C and Figure S4C–D). The Y414S/L/R, D417N, L419A, S463T/Y/F, Y469N and R470K caused marked binding reductions to the HKU1 RBD, relative to wildtype TMPRSS2, consistent with the compatibility probably scores (Figure 4C and Figure S4C–D). Furthermore, the HKU1 RBD bound much more weakly to uncleaved, relative to cleaved, TMPRSS2. These findings possibly narrow the TMPRSS2 species tropism of HKU1 as mammalian TMPRSS2s frequently harbor D417N and Y469N substitutions sometimes accompanied by a mutation of residue Y414 (Figure 4B). L419 and S463 are not conserved for non-mammalian TMPRSS2s and R470 is only partially conserved among reptile/bird TMPRSS2s and not conserved for TMPRSS2s found in amphibians and other vertebrates. The combination of these residue substitutions along with the Y414 and Y469 mutations, are expected to limit the ability of the HKU1 RBD to engage non-mammalian TMPRSS2 orthologs.

Based on these binding data, we hypothesized that TMPRSS2 orthologs from many Primates, Rodents, and Artiodactyls, as well as some Lagomorphs and Chiropterans orthologs, may permit HKU1 S-mediated entry. Using a vesicular stomatitis virus (VSV) pseudotyped with HKU1 isolate N1 S, we found that transient transfection of Primate (including Human, Chimpanzee, Capuchin, and Marmoset), Rodent (including Natal Multimammate mouse, Brown Rat, and Hamster), Artiodactyl (Camel, Alpaca, and Cow), Lagomorph (Pika), and Chiropteran (Flying Fox) TMPRSS2s promote HKU1 S-mediated entry into HEK293T cells (Figure 4D and Figure S6). Most of these non-human TMPRSS2 orthologs harbor the D417N residue substitution, suggesting that this mutation alone does not prevent HKU1 S-mediated entry. Although Brown Rat TMPRSS2 (possessing the D417N substitution) and Hamster TMPRSS2 (harboring the S412N, D417N and Y469L mutations) rendered cells susceptible to HKU1 S VSV, House Mouse TMPRSS2 (possessing D417N and Y469L mutations) did not (Figure 4D and Figure S6). The S412N House Mouse TMPRSS2 mutant promoted detectable HKU1 S VSV entry into target cells, indicating that this residue substitution explains (at least in part) that Hamster TMPRSS2 supports cell entry but not House Mouse TMPRSS2 (Figure 4D and Figure S6). This finding is further supported by the greater binding of the mouse S441A/S412N TMPRSS2 ectodomain to the HKU1 RBD, as compared to the S441A mouse TMPRSS2 ectodomain, and suggests that S412N could act epistatically in the hamster and mouse TMPRSS2 backgrounds to enhance HKU1 RBD binding (Figure 4C). The D417N/Y469N residue substitutions likely account for the reduced HKU1 S VSV entry into cells promoted by Ferret TMPRSS2, relative to human TMPRSS2, which is further compounded by low cell-surface expression in the case of the Horseshoe Bat TMPRSS2 (Figure 4C–D and Figure S6). The combination of the Y414R and Y469N residue substitutions in African Green Monkey TMPRSS2 explains that it is the sole Primate ortholog evaluated in our panel that do not support HKU1 S VSV entry into cells (Figure 4C–D and Figure S6). Low HKU1 S VSV entry into cells transiently transfected with Mole Rat TMPRSS2 might result from the L430I mutation in the binding site, possibly clashing sterically with the HKU1 RBD Y528 residue. Chicken TMPRSS2 minimally supported HKU1 S VSV entry, while Chameleon and African clawed frog TMPRSS2s did not lead to detectable entry, concurring with the presence of the D417N, L419A, S463T, Y469N or R470K mutations in these orthologs with possible additional negative contribution of the A468K substitution in Chameleon TMPRSS2 (Figure 4C–D and Figure S6). Collectively, our data explain the efficient HKU1 use of human TMPRSS2 as entry receptor and reveal that several TMPRSS2 orthologs support HKU1 S-mediated entry into cells.

### Functional consequences of TMPRSS2 site mutants in humans

We found several low frequency TMPRSS2 single nucleotide polymorphisms (SNPs) at residue positions involved in HKU1 binding and several of them correspond to missense mutations also observed in TMPRSS2 paralogs and orthologs (Figure 4B and Data S1–S2). Among the SNPs we characterized experimentally, S463F and R470K almost entirely abrogated binding (Figure 4C, Figure S5 and Data S1–S2). R470K (​​allele frequency: 6.1×10^−7^) possibly disrupts pi-stacking interactions with the HKU1 RBD R517 side chain^58^ whereas S463F (​​allele frequency: 2.1×10^−6^) would sterically hinder HKU1 RBD binding to TMPRSS2 given that none of the energetically favored phenylalanine side chain rotamers at this position would be sterically compatible with the binding interface in our cryoEM structure. In summary, although several TMPRSS2 SNPs map to the site of HKU1 attachment, some of them possibly reducing susceptibility to HKU1 infection (e.g. S463F and R470K), their low frequency is unlikely to majorly impact HKU1 transmission in the human population.

Residues involved in binding to the HKU1 RBD markedly diverge across human type 2 transmembrane serine proteases with G462 being the sole position strictly conserved (Figure 4B). Members of the TMPRSS protease subfamily harbor non-conservative mutations at several positions involved in HKU1 binding relative to TMPRSS2. This includes L419A (TMPRSS5), S463T/Y (hepsin, TMPRSS4, TMPRSS13, and enterokinase), and Y469N (TMPRSS3, TMPRSS5, TMPRSS13, and enterokinase), which we found to abrogate or severely dampen TMPRSS2 binding to the HKU1 RBD (Figure 4B–C and Figure S5). These data therefore rationalize the lack of HKU1 S-mediated fusion observed in the presence of TMPRSS3, TMPRSS4, and TMPRSS13^37^ and explain the HKU1 specificity for TMPRSS2.

### Glycan shielding and conformational masking mediate immune evasion

**Figure 5.**
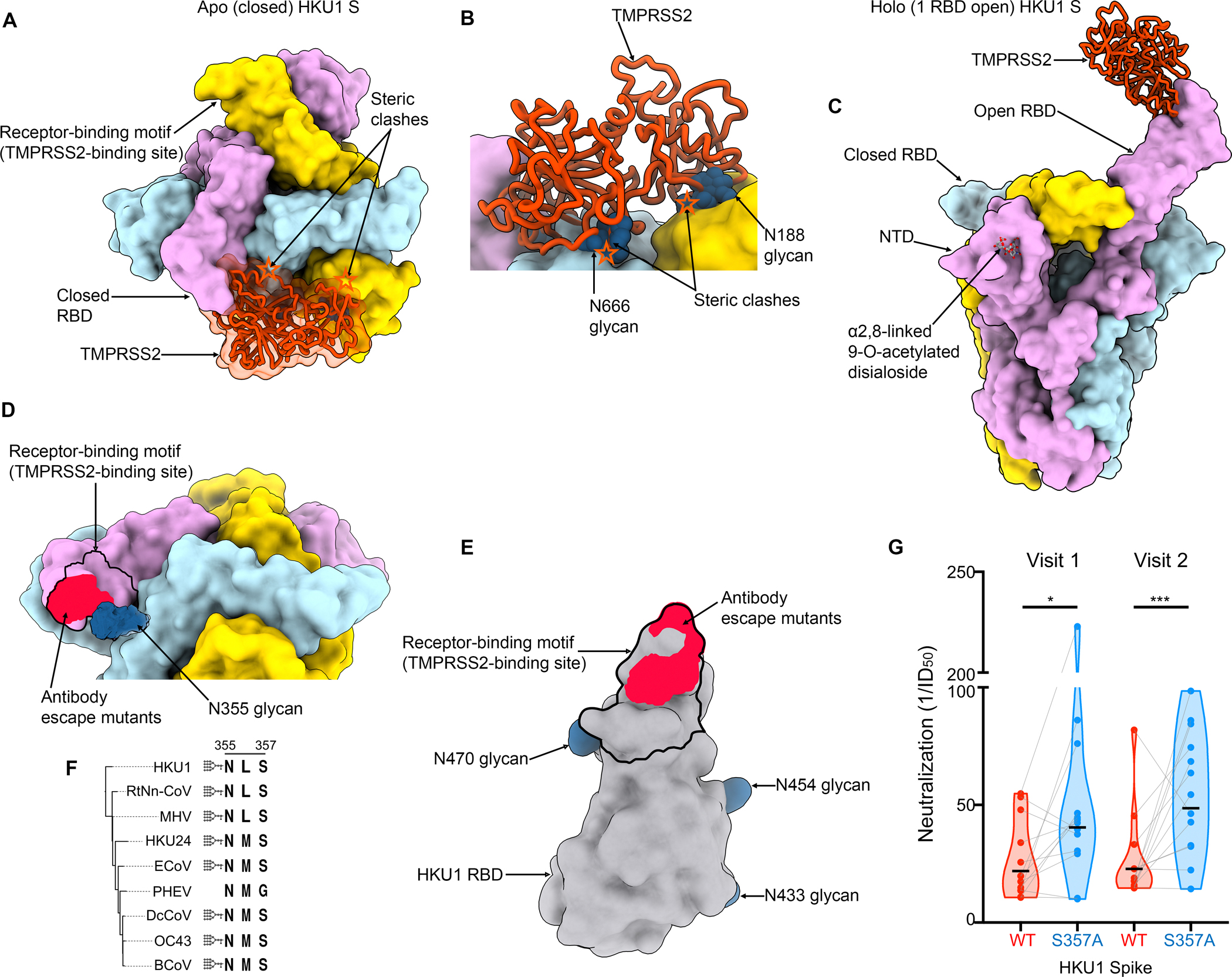
Glycan shielding and conformational masking mediate HKU1 immune evasion (A-B) Composite model obtained by superimposing the TMPRSS2 (orange)-bound HKU1 RBD cryoEM structure onto the apo closed HKU1 S trimer structure (PDB 8OHN)^38^ rendered as a surface with each protomer colored distinctly viewed in two orthogonal orientations. In the closed trimer conformation, TMPRSS2 binding would be hindered by steric clashes (orange stars) with neighboring protomers, including with N-linked glycans N188, N355, N666 (shown as blue spheres). (C) Composite model obtained by superimposing the TMPRSS2 (orange)-bound HKU1 RBD cryoEM structure onto the α2,8-linked 9-O-acetylated disialoside-bound HKU1 S trimer with one open RBD (PDB 8OPN)^38^ engaging the TMPRSS2 receptor. (D) Zoomed-in view of the apo closed HKU1 S trimer structure (PDB 8OHN)^38^ showing the unsharpened cryoEM density for the N355 glycan partially masking the receptor-binding motif (black outline) and the site of vulnerability to the mHKUS-2 and -3 neutralizing antibodies^55^ (neon red). The extent of the N355 glycan-mediated glycan shielding is expected to be even greater than shown as only the first three monosaccharides are resolved in the map. (E) HKU1 RBD rendered as a grey surface with the TMPRSS2 footprint delineated with a black outline and the escape mutants (D511, H512, W515, R517) identified for the neutralizing antibodies mHKUS-2 and -3 shown in neon red. The HKU1 RBD N-linked glycans are rendered as blue surfaces. (F) Neighbor-Joining phylogram of embecovirus S glycoproteins focused on the HKU1 N355 glycosylation site. Included S sequences are HKU1 (AZS52618.1), RtNn-CoV (RtNn-CoV/SAX2015, ATP66762.1), mouse hepatitis virus (MHV, C0KYY9), HKU24 (A0A866W1F1), Equine coronavirus (ECoV, E5RPZ2), Porcine hemagglutinating encephalomyelitis virus (PHEV, A0A1Z2WUW0), Dromedary Camel coronavirus (DcCoV, HKU23, A0A291L0R6), OC43 (P36334) and bovine coronavirus (BCoV, P15777). (G) Neutralization of VSV pseudotyped with wildtype isolate N1 HKU1 S (WT) or the N355 glycan knockout (S357A) mutant by serum antibodies elicited by HKU1 infection. Visits 1 and 2 correspond to blood draws at the time of PCR-positive testing and 1–2 months later, respectively^94,95^. Neutralization values were compared by two-way ANOVA with the Geisser-Greenhouse correction and Tukey’s multiple comparison test; * corresponds to a P value of 0.02, while *** corresponds to a P value of 0.0007.

Although the HKU1 RBM protrudes out from the closed S trimer, leading to increased solvent exposure relative to sarbecovirus RBMs^12,54^, TMPRSS2 binding would be precluded due to steric hindrance involving both proteinaceous and oligosaccharide moieties from neighboring protomers (Figure 5A–B). Sialoglycan receptor binding, which has recently been shown to promote RBD opening^38^, would relieve these steric constraints and enable TMPRSS2 attachment to an RBD in the open conformation (Figure 5C). Similar to other coronaviruses, this cascade of events is expected to lead to fusion of the viral and host membranes to promote genome delivery to a target cell and initiation of infection^54,59,60^.

The HKU1 glycan at position N355 from a neighboring RBD masks the RBM (Figure 5D), as observed in an apo closed HKU1 S structure^38^, sterically limiting RBM accessibility in this conformation. As a result, residues D511, H512, W515 and R517, which map to the RBM and contribute to the epitopes recognized by the previously described mHKUS-2 and -3 neutralizing monoclonal antibodies^55^, are also partially masked by the HKU1 N355 glycan which would reduce targeting by this class of antibodies (Figure 5D–E). The overlap between the RBM and the epitopes of the mHKUS-2 and -3 antibodies suggest that their primary mechanism of neutralization is through direct competition with HKU1 attachment to the TMPRSS2 host receptor, in line with prior studies of potent sarbecovirus and merbecovirus antibodies^54,61–66^. Given the conservation of this glycan in most embecovirus S glycoproteins (Figure 5F), similar masking is anticipated to occur, as confirmed in the prefusion OC43 S structure^31^ although the role of the topological equivalent of the RBD (domain B) remains unclear for many members of this subgenus. Collectively, our data suggest that HKU1 relies on conformational masking and glycan shielding to balance conflicting requirements for immune evasion and receptor engagement, ultimately modulating viral fitness.

## DISCUSSION

HKU1 genome sequences are divided into three genotypes designated A, B and C with evidence of recombination among them^5,9^. The S glycoproteins of genotypes A and B share 85% amino acid sequence identity whereas that of genotypes B and C are almost identical^37^. Furthermore, HKU1 RBDs are highly divergent with ~74% amino acid sequence identity between the HKU1 isolate N1 RBD (genotype A) and the isolate N2 RBD (genotype B) or the isolate N5 RBD (genotype C). This magnitude of sequence divergence is comparable to that observed among the RBDs of distinct sarbecovirus clades^67–69^ and may participate in explaining that protective immunity to seasonal coronaviruses is of short duration due to antigenic drift^70,71^. The presence of epitopes targeted by neutralizing antibodies in the RBM^55^ along with the N355 glycan shielding of the RBM in the closed S trimer suggest that RBM-directed antibodies are making a key contribution to neutralizing activity, as is the case for other coronaviruses^62^. These findings may explain the observed polymorphism of TMPRSS2-interacting residues among HKU1 isolates and are reminiscent of observations made for SARS-CoV-2^62,72,73^ and 229E^71,74^. Our structural data therefore provide a molecular blueprint to follow evolution of RBM residues and their impact on receptor binding and immune evasion. Furthermore, these findings suggest that removal of the N355 glycan might be a suitable vaccine design strategy to improve the potency of antibody responses elicited by HKU1 S and for most viruses from the embecovirus subgenus. Although the OC43 domain B is not known to be a functional RBD, neutralizing antibodies recognizing this region were described^75^, underscoring the possible role of this conserved embecovirus glycan for immune evasion. Since HKU1 recognizes 9-O-acetylated sialosides using the S NTD^32,34,38,76^, similar to OC43 and bovine coronavirus^22,31^, the isolation of a neutralizing antibody competitively inhibiting sialoside binding to the OC43 NTD^75^ suggests that this mechanism of action is likely conserved against many, if not all, lineage A β-coronaviruses attaching to cell-surface carbohydrates. Blocking sialoside receptor binding to the MERS-CoV NTD has also been described to mediate viral neutralization of this highly pathogenic human coronavirus^77^. Future studies will elucidate the importance and possible synergy of these different types of antibodies, along with that of non-neutralizing antibodies, for in vitro neutralization and in vivo protection against these viruses.

Rapid antigenic evolution, masking of epitopes and exposure of epitopes targeted by non-neutralizing antibodies are immune-evasion strategies widely used by viruses. Coronavirus S trimers can adopt at least two distinct conformations of their RBDs enabling to mask or expose the RBM in the closed and open states, respectively. Although the SARS-CoV-2, SARS-CoV-1 and MERS-CoV S trimers appear to populate this conformational landscape spontaneously^12,54,78,79^, most coronavirus S trimers appear to preferentially adopt a closed conformation^21,31,53,60,69,80–84^. Recent work showed that α2,8-linked 9-O-acetylated disialosides binding to the HKU1 S NTD induces conformational changes leading to RBD opening^38^, which our data reveal to expose the TMPRSS2-binding site. This elegant mechanism would allow to ensure proper spatial and temporal coordination of these conformational changes, ensuring that RBD opening occur upon attachment to a target cell, thereby balancing the conflicting requirements for receptor binding to initiate infection (which requires RBD opening) and immune evasion through masking of a key site of vulnerability (RBM) recognized by neutralizing antibodies (which requires RBD closing).

Although HKU1 has been proposed to have originated in rodents, due to phylogenetic clustering with rodent coronaviruses^85^, evidence supporting or invalidating this hypothesis is still lacking. We identified the molecular determinants of HKU1 utilization of TMPRSS2 and revealed that TMPRSS2 orthologs from at least five mammalian orders (Primate, Rodent, Artiodactyl, Lagomorph, and Chiropteran) support HKU1 S-mediated entry into cells. These data are compatible with a possible rodent origin of HKU1 and the putative involvement of the species identified here as reservoir or intermediate hosts. Several of these species have already been implicated in zoonotic transmission of other viruses including Natal Multimammate mice (Lassa Fever Virus), Brown rat (Hantavirus), Flying Fox (Nipah and Hendra viruses), Chimpanzee (HIV-1), and Camel (MERS). Considering the compatibility with HKU1 S-mediated entry into cells of several TMPRSS2 orthologs from species used as animal models (e.g. Hamster, Chimpanzee, Marmoset)^66,86–91^, our data suggest a possible path to develop an HKU1 challenge model to study pathogenicity, correlates of protection and the effectiveness of countermeasures.

### Limitations of the study

We note that host tropism is a complex process involving multiple other factors than receptor recognition, including proteolytic spike activation and innate immune antagonism, for a successful infection to occur.

## STAR METHODS

### Lead contact

Further information and requests for resources and reagents should be directed to and will be fulfilled by the lead contact David Veesler (dveesler@uw.edu)

### Materials availability

All datasets generated and information presented in the study are available from the corresponding authors on reasonable request. Materials generated in this study can be available on request and may require a material transfer agreement.

### Data and code availability

The cryoEM map and atomic model have been deposited to the EMDB and PDB with accession codes EMD-43224 and PDB 8VGT.

### EXPERIMENTAL MODEL AND SUBJECT DETAILS

#### Cell lines

Cell lines used in this study were obtained from the American Type Culture Collection (ATCC, for HEK293T) or Thermo Fisher Scientific (ExpiCHO-S cells and Expi293F cells).

#### Constructs

The membrane-anchored HKU1 isolate N1 S (ref. seq. YP_173238, genotype A) with a 21 amino acid C-terminal deletion and six residues flexible linker followed by C-terminal HA tag were codon-optimized, synthesized, and inserted the pcDNA3.1(+) vector by Genscript. The HKU1 RBD constructs encoding S residues 320–614 of the wildtype isolate N1 (ref. seq. YP_173238, genotype A) and the H488A, E505A, V509A, L510A, W515A, R517A and Y528A HKU1 RBD interface mutants, containing N-terminal mu-phosphatase secretion signal peptide, with or without an avi tag followed by three residues flexible linker and C-terminal octa-histidine tag were codon optimized, synthesized, and inserted into the pcDNA3.1(+) vector by Genscript. The membrane-anchored TMPRSS2 and TMPRSS2 ectodomain constructs were codon-optimized, synthesized, and inserted into a pCMVR vector. TMPRSS2 ectodomain constructs were further mutated by In-Fusion Assembly (Takara Bio). All TMPRSS2 sequences are listed in Table S1.

The SARS-CoV-2 S 2P (Wuhan-Hu-1) ectodomain construct^12^ harbors an N-terminal mu-phosphatase secretion signal peptide, S1/S2 cleavage site R682S/R683G/R685G mutations, K986P/V987P mutations, one residue linker followed by C-terminal hexa-histidine tag and foldon trimerization domain was codon-optimized, synthesized, and inserted the pCMV vector by Genscript.

#### Generation of pseudoviruses

To produce pseudoviruses for entry and neutralization assays, HEK293T cells were seeded in Dulbecco’s Modified Eagle Medium (DMEM) enriched with 10% Fetal bovine serum (FBS, Hyclone), 1% PenStrep (100 I.U./mL penicillin and 100ug streptomycin) (Gibco 15140-122) at the appropriate density to yield 80% confluency in polylysine-coated 100 mm cell culture dishes and placed in an incubator at 37°C with 5% CO_2_. After 18–22 hr incubation, cells were washed with OPTI-Minimum Essential Media (Opti-MEM, Life Technologies). 24μg of either the wildtype HKU1 isolate N1 (genotype A) S or the S357A mutant S plasmids were prepared in 1.5mL of OPTI-MEM and combined with 60μL of Lipofectamine 2000 (Life Technologies) diluted in 1.5mL of OPTI-MEM and incubated at room temperature for 15–20 min. The mixture was added to the HEK293T cells which were placed for 2 hours in an incubator at 37°C with 5% CO_2_ after which 2mL of DMEM enriched with 20% FBS and 2% PenStrep (200 I.U./mL penicillin and 200ug streptomycin) was added to the transfected cells and incubated overnight. The following day, cells were washed with DMEM and transduced with VSVΔG/Fluc and incubated for 2 hours at 37°C with 5% CO_2_. After washing with DMEM, medium supplemented with anti-VSV-G antibody (I1-mouse hybridoma supernatant diluted to 1:25 from CRL-2700, ATCC) was added to the cells to reduce background from the parental virus and an additional incubation at 37°C with 5% CO_2_ was performed overnight. The next day, the supernatant from the cells was harvested from the 100 mm dishes, further clarified by centrifugation at 3,000×g for 10 minutes, filtered (0.45μm), and concentrated 10 times by using centrifugal devices with 30 kDa cutoff membranes. Pseudoviruses were then aliquoted and frozen at −80°C until used.

#### Pseudovirus neutralization and entry assays

HEK293T cells were cultured in DMEM with 10% FBS (Hyclone), 1% PenStrep (100 I.U./mL penicillin and 100 μg streptomycin) and placed in cell-culture grade, polylysine-coated 100 mm plates overnight in an incubator at 37°C with 5% CO_2_. The following day, when cells reached 90% confluency, they were transfected with 4 μg of TMPRSS2 and incubated for 5 hours. The cells were subsequently trypsinized, counted and reseeded at ~40,000 cells per well in poly-lysine-coated 96 well plates and placed in an incubator at 37°C with 5% CO_2_ overnight. The next day, a half-area 96-well plate was prepared with a 1:3 serial dilution of sera in DMEM in 22 μL final volume. 22 μL of pseudovirus was then added to each well and incubated at room temperature for 30–45 min. The media was removed from transfected HEK293T cells and 40 μL of the sera/pseudovirus mixture was added to the cells and incubated for 2 h at 37°C with 5% CO_2_ before adding 40 μL of 20% FBS and 2% PenStrep (200 I.U./mL penicillin and 200 μg streptomycin) containing DMEM. No sera were used for assessment of entry with the panel of TMPRSS2 orthologs. Following 18–22 hour incubation, 40 μL of One-GloEX (Promega) was added to the cells and incubated in the dark for 5 min prior to reading on an Agilent BioTek Neo2 plate reader. Relative luciferase units were plotted and normalized in Prism (GraphPad) using a zero value of cells alone and a 100% value of 1:2 virus alone. Nonlinear regression of log(inhibitor) vs. normalized response was used to determine ID_50_ values from curve fits. At least two biological replicates with two distinct batches of pseudovirus were conducted for each serum sample.

#### Flow cytometry

Flow cytometry was utilized to quantify expression levels of different TMPRSS2 plasmids on the surface of HEK293T cells. HEK293T cells were cultured in DMEM enriched with 10% FBS (Hyclone), 1% PenStrep (100 I.U./mL penicillin and 100ug streptomycin) and plated in cell-culture grade, 6-well Corning flat bottom plates (Fisher #3516). The plates were then kept overnight in an incubator at 37°C with 5% CO_2._ After reaching ~90% confluency the following day, cells were then washed twice with unenriched DMEM and a final volume of 2.5mL of enriched DMEM was added to the cells. 4 μg of each TMPRSS2 plasmid were prepared in 250 μL of Opti-MEM and combined with 10 μL of Lipofectamine 2000 diluted in 240 μL of Opti-MEM and incubated at room temperature for 10–15 minutes. The mixture was subsequently added to the HEK293T cells (bringing the final volume on the plate to 3mL) and cells were then incubated overnight at 37°C with 5% CO_2_. The next day, the transfected cells were washed twice with cell culture grade PBS pH 7.4 and then dissociated on a shaker at 37°C in 500 μL of enzyme-free cell dissociation buffer (Gibco #13151014) for 10 minutes. Cells were then filtered through a cell-strainer capped tube, diluted to ~1×10^6 cells per mL and added to a pyramid bottom 96-well plate. Cells in the plate were centrifuged and the supernatant discarded. Cells were resuspended in PBS containing 10 μg/mL of Monoclonal ANTI-FLAG^®^ M2 antibody (Sigma F3165-1MG) and incubated at 4C for ~25 minutes. Cells were washed 3 times with PBS before adding 5 μg of Goat anti-Mouse IgG Fc Secondary Antibody, FITC (ThermoFisher 31547) After ~25 minutes at 4°C, cells were washed three times with PBS. To fix the cells, 100 μL of 2% paraformaldehyde was added to each well and incubated at 4°C for 15 minutes. Cells were then washed twice with PBS prior to being resuspended in 50 μL of fresh PBS. Cells were transferred into tubes with 450μL of PBS, kept on ice, shielded from direct light prior to being examined using a BD FACSAriaIII and FACSDiva for acquisition and FlowJo 10.8.2 for analysis. Cells were gated on singleton events and 3,500 to 8,000 singleton events were collected per sample. TMPRSS2 expression levels were quantified using the geometric mean FITC intensity measured for the singleton events.

#### Recombinant glycoprotein production

The SARS-CoV-2 S 2P ectodomain, HKU1 RBDs or TMPRSS2 ectodomains were produced and purified using Expi293F or ExpiCHO-S cells. Expi293F cells were grown to a density of 3 × 10^6^ cells/mL and transfected using the ExpiFectamine 293 Transfection Kit (ThermoFisher Scientific) and expression carried out for 3 to 5 days post-transfection at 37°C with 8% CO_2_. ExpiCHO-S cells were grown to a density of 6 × 10^6^ cells/mL and transfected using the ExpiFectamine CHO Transfection Kit (ThermoFisher Scientific) and expression carried out for 7–10 days post-transfection at 37°C with 8% CO_2_. The SARS-CoV-2 S 2P ectodomain and HKU1 RBDs were purified from clarified supernatants using HisTrap HP affinity columns (Cytiva) and washed with ten column volumes of 10 mM imidazole, 25 mM sodium phosphate pH 8.0, and 300 mM NaCl before elution with two column volumes of 300 mM imidazole, 25 mM sodium phosphate pH 8.0, and 300 mM NaCl. The purified HKU1 RBD proteins were buffer exchanged into 20 mM sodium phosphate pH 8 and 100 mM NaCl or 50 mM Tris pH 8.0 and 150 mM NaCl. The HKU1 RBDs were biotinylated using the biotin ligase (BirA) reaction kit (Avidity) following the manufacturer’s protocol. Biotinylation was carried out at room temperature for 30 minutes followed by incubation for 10 hours at 4°C. TMPRSS2 constructs were purified using TALON metal affinity resin (Takara Bio) and washed with 100 column volumes of 5 mM imidazole, 50mM 4-(2-hydroxyethyl)-1-piperazineethanesulfonic acid (HEPES) pH 7.5, and 150mM NaCl prior to elution with 600 mM imidazole, 50mM HEPES pH 7.5, and 150mM NaCl. Purified TMPRSS2s were diluted to 0.3 mg/ml and then digested with a 1/500 dilution of enterokinase (EKmax, ThermoFisher) for 16 hours at room temperature or 1 hour at 37 °C to convert the zymogen in active enzyme, remove the SUMO tag and the His tag. Digested TMPRSS2 was then passed through fresh TALON metal affinity resin to remove enterokinase and the cleaved His tag. Active TMPRSS2 with the DS mutation, SUMO tag, and restored N249 glycan activated itself during expression and purification, and did not require enterokinase activation. Purified HKU1 RBDs and TMPRSS2s – except for TMPRSS2 used for Figure 4D – were then further purified by size exclusion chromatography using a Superdex 200 Increase 10/300 GL column (Cytiva) and concentrated using centrifugal filters (Amicon Ultra) before being flash frozen.

#### Biolayer interferometry

Biotinylated HKU1 wildtype (isolate N1, genotype A) RBD and H488A, E505A V509A, L510A, W515A, R517A and Y528A HKU1 RBD interface alanine mutants were diluted to concentrations of 0.002 mg/mL in modified kinetics buffer (1% BSA, 0.06% Tween 20, 1x Cold Spring Harbor PBS pH 7.4) and loaded onto pre-hydrated streptavidin biosensors to a 1 nm total shift. The loaded tips were dipped into 100 nM TMPRSS2 (harboring the S441A residue substitution and the C379-T447C disulfide bond) for 200 or 300 seconds followed by dissociation in modified kinetics buffer for 500 seconds. Analysis of TMPRSS2 interface alanine mutants was carried out similarly but with constructs harboring S441 and the C379-T447C disulfide bond. Binding curves were plotted using Graphpad Prism 10.1.2. The area under the curves were determined by summation of the binding response values for each reading, with a limit of binding detection of 0.05 nm. Sums were normalized to WT, as was the limit of detection which corresponded to 2 ± 0.5 % (the practical limit of binding detection was set to two standard deviations above this value, i.e. 3.1 % of WT binding).

#### TMPRSS2 residue conservation analysis and predicted binding compatibility

TMPRSS2 sequences were retrieved from the NCBI. In MEGA11^96^, these sequences were aligned using the MUSCLE algorithm and a NJ tree was constructed with 1000 bootstrap replications. Using Microsfot Excel, each residue in the alignment was scored for conservation compared with human TMPRSS2 using the BLOSUM 62 matrix. A predicted binding compatibility matrix was constructed by calculating the ProteinMPNN^57^ scores (averaged from 30 runs) for each TMPRSS2 residue in the HKU1 RBD-bound TMPRSS2 structure, and then subtracting the ProteinMPNN scores (averaged from 30 runs) for each TMPRSS2 residue after the HKU1 RBD was removed from the coordinate file (thus controlling for residues predicted to generally stabilize TMPRSS2 rather than the HKU1 RBD-binding interface). Each residue in the alignment was then scored for predicted binding compatibility using this matrix. The mean conservation score or predicted binding compatibility score for each residue in the alignment was then calculated from four subsets of the alignment: mammals, reptiles and birds, amphibians, and other vertebrates. The mean conservation score or predicted binding compatibility score for all residues that engage HKU1-RBD was then calculated for each TMPRSS2 ortholog.

#### TMPRSS2 enzymatic activity assay

Peptidase assays were performed with Boc-QAR-AMC substrate (GlpBio) in black half area 96 well plates (Greiner Bio-One Fluotrac) in 100 μl reaction volumes using a Agilent BioTek Neo2 Microplate Reader at 22 °C, monitoring fluorescence every two minutes over 30 minutes at 341 nm excitation and 441 nm emission. The slope of the fluorescence curve over the first 10 minutes (corresponding to <5 % substrate conversion) was used for velocity calculations. The AMC concentration was calculated using standard curves at each substrate and HKU1 RBD concentration, to correct for the inner filter effect. For determination of TMPRSS2 enzyme kinetics, serial dilutions of Boc-Gln-Ala-Arg-AMC substrate were reacted with 6.8 nM TMPRSS2. For determination of the *K*_*I*_ of the HKU1 RBD, 5 nM TMPRSS2 was reacted with serial dilutions of Boc-QAR-AMC substrate in the presence of 215, 72, or 24 nM HKU1 RBD (TMPRSS2 was incubated for 2 minutes with the HKU1 RBD at 22 °C prior to the addition of substrate). Velocities were plotted and fit using GraphPad Prism (v10.1.1).

#### SARS-CoV-2 S cleavage assay

Spike cleavage assays were performed with SARS-CoV-2 S-2P ectodomain in 20mM phosphate pH 8.0 and 100mM NaCl incubated with 500nM TMPRSS2 in 50 mM HEPES pH 7.5 150mM NaCl. Samples were diluted in 50 mM HEPES pH 7.5 150mM NaCl, mixed, and incubated at 37°C with 300 rpm shaking. Reactions were stopped at various time points by mixing with 4x gel loading buffer containing 200mM Tris pH 6.8, 8% SDS, 20% β-mercaptoethanol, 40% Glycerol, 0.2% Bromophenol blue. The HKU1 RBD inhibition assays were performed by incubating the HKU1 RBD (without avi tag) with TMPRSS2 (harboring the N-terminal SUMO fusion and the C379-T447C disulfide bond) in 50 mM HEPES pH 7.5 150mM NaCl for 5 minutes at 37°C prior to adding SARS-CoV-2 S 2P and incubating at 37°C with shaking at 600 rpm for 15 minutes prior to stopping the reaction by mixing with 4x gel loading buffer containing 200mM Tris pH 6.8, 8% SDS, 20% β-mercaptoethanol, 40% Glycerol, 0.2% Bromophenol blue. Samples were then analyzed by SDS-PAGE.

#### Single nucleotide polymorphism (SNP) analysis

The SNP information was extracted from two human population allele frequency database, GnomAD (version 4) and Regeneron’s Million Exome Variant database. For GnomAD v4, we utilized the allele frequencies of short genetic variations from the exome sequencing data of 730,947 individuals. These genetic variations have passed the QC (i.e. “FILTER” column in the VCF = “PASS”). GnomAD v4 provided the functional consequences of the genetic variations from the VEP tool. We obtained the predicted deleteriousness of mutations from AlphaMissense^97^. For Regeneron’s Million Exome Variant data, we utilized the allele frequencies of short genetic variations from the exome sequencing data of 983,578 individuals. We observed a total of 49 short genetic variations in the GnomAD v4 and the Regeneron’s Million Exome Variant databases.

#### CryoEM sample preparation, data collection and data processing

Complex formation was performed by mixing a 1:1.2 molar ratio of the cleaved human TMPRSS2 ectodomain (harboring the S441A residue substitution and a C379-T447C disulfide bond) with the HKU1 isolate N1 RBD (residues 320–614 with C-terminal octa-histidine tag and no avi tag) before incubation for 1 hour at 4°C. CryoEM grids of the complex were prepared using three separate methods and data were combined during data processing. For the first dataset, 3 μL of 1.1 mg/mL or 0.5 mg/mL of complex were loaded onto freshly glow discharged R 2/2 UltrAuFoil grids^98^ prior to plunge freezing using a Vitrobot Mark IV (ThermoFisher Scientific) with a blot force of 0 and 6 sec blot time at 100% humidity and 22°C. 6,681 movies from UltrAuFoil grids with 1.1 mg/mL protein complex were collected with a defocus range comprised between −0.2 and −3 μm and stage tilt angle of 0° and 20°^99^. 3,820 movies from UltrAuFoil grids with 0.5 mg/mL protein complex were collected with a defocus range comprised between −0.2 and −3 μm and stage tilt angle of 30° and 45°. For the second dataset, 3 μL of 1 mg/mL complex was added to the glow discharged side of R 2/2 UltrAuFoil grids and 1μL was added to the back side before plunging into liquid ethane using a GP2 (Leica) with 6 sec blot time. 9,883 movies were collected with a defocus range comprised between −0.2 and −3 μm and stage tilt angle of 0° and 30°. For the third dataset, 3 μL of 8 mg/ml complex with 3 mM 3-[(3-Cholamidopropyl)dimethylammonio]-2-hydroxy-1-propanesulfonate (CHAPSO) or 0.01% fluorinated octyl-maltoside (FOM) (Anatrace) were applied onto freshly glow discharged R 2/2 UltrAuFoil grids prior to plunge freezing using a vitrobot MarkIV (ThermoFisher Scientific) with a blot force of 0 and 5 sec blot time at 100% humidity and 22°C. 6,822 and 1,859 movies were collected from UltrAuFoil grids with CHAPSO and FOM detergents, respectively, with a defocus range comprised between −0.2 and −3.5 μm. The data were acquired using an FEI Titan Krios transmission electron microscope operated at 300kV and equipped with a Gatan K3 direct detector and Gatan Quantum GIF energy filter, operated in zero-loss mode with a slit width of 20 eV. Automated data collection was carried out using Leginon^100^ at a nominal magnification of 105,000× with a pixel size of 0.835 Å. The dose rate was adjusted to 9 counts/pixel/s, and each movie was acquired in counting mode fractionated in 100 frames of 40 ms. Movie frame alignment, estimation of the microscope contrast-transfer function parameters, particle picking, and extraction were carried out using Warp^101^. Particles were extracted with a box size of 168 pixels with a pixel size of 1.67Å. Two rounds of reference-free 2D classification were performed using CryoSPARC^102^ to select well-defined particle images from each dataset, which were subsequently combined. Initial model generation was done using ab-initio reconstruction in cryoSPARC and used as references for a heterogenous 3D refinement in cryoSPARC. Particles belonging to classes with the best resolved HKU1 RBD and TMPRSS2 density were selected. To improve particle picking further, we trained the Topaz^103^ picker on Warp-picked particle sets belonging to the selected classes after heterogeneous 3D refinement. The particles picked using Topaz were extracted and subjected to 2D classification using cryoSPARC, which improved the number of unique 2D views. The two different particle sets picked from Warp and Topaz were merged and duplicate particle picks were removed using a minimum distance cutoff of 60Å. After two rounds of heterogeneous refinements and removal of junk particles, 3D refinement was carried out using non-uniform refinement with per-particle defocus refinement in cryoSPARC^104^ and the particles were transferred from cryoSPARC to Relion using pyem (https://github.com/asarnow/pyem) to be subjected to the Bayesian polishing procedure implemented in Relion^105^ during which particles were re-extracted with a box size of 280 pixels and a pixel size of 1.0 Å. Subsequent 3D refinement used non-uniform refinement along with per-particle defocus refinement in cryoSPARC to yield the final reconstruction at 2.9 Å resolution comprising 810,357 particles. Local resolution estimation, filtering, and sharpening were carried out using cryoSPARC. Reported resolutions are based on the 0.143 gold-standard Fourier shell correlation (FSC) criterion and Fourier shell correlation curves were corrected for the effects of soft masking by high-resolution noise substitution^106,107^.

#### Model building and refinement

UCSF Chimera^108^ was used to rigid-body dock models into the sharpened cryoEM map and adjustments and refinement were carried out with Coot^109^ and Rosetta^110,111^ using sharpened and unsharpened maps. Validation used Molprobity^112^, Phenix^113^ and Privateer^114^.

## Supplementary Material

FigS3 Figure S3, related to Figure 2. Comparison of the isolated HKU1 RBD and TMPRSS2 structures to the TMPRSS2-bound HKU1 RBD cryoEM structure. (A) Ribbon diagram of the cryoEM structure of the HKU1 RBD (purple) bound to the human TMPRSS2 ectodomain superimposed to the crystal structure of the apo HKU1 RBD (blue, PDB 5KWB). The TMPRSS2 ectodomain is omitted for clarity. (B) Ribbon diagram of the cryoEM structure of the human TMPRSS2 ectodomain (orange) bound to the HKU1 RBD superimposed to the crystal structure of the nafamostat-bound TMPRSS2 (green, PDB 7MEQ). The HKU1 RBD is omitted for clarity.

FigS4 Figure S4, related to Figure 2 and 4. Evaluation of TMPRSS2 binding to biotinylated HKU1 RBD immobilized on biolayer interferometry SA biosensors. (A) Two panels show baseline-subtracted response curves for the first (left panel) and second (right panel) biological replicates of TMPRSS2 S441A binding to HKU1 RBD mutants (related to Figure 2). Biotinylated HKU1 isolate N1 RBD-loaded SA tips were dipped into 100 nM TMPRSS2 S441A for 200 seconds followed by dissociation for 400 seconds. (B) Three panels show baseline-subtracted response curves for the first, second, and third biological replicates of TMPRSS2 mutants binding to the HKU1 RBD (related to Figure 2). For the first and second biological replicate (first two panels), biotinylated HKU1 isolate N1 RBD-loaded SA tips were dipped into 100 nM TMPRSS2 mutants for 200 seconds followed by dissociation for 400 seconds. For the third biological replicate (last panel), biotinylated HKU1 isolate N1 RBD-loaded SA tips were dipped into 100 nM TMPRSS2 mutants for 300 seconds followed by dissociation for 500 seconds. (C) Baseline-subtracted response curves for the first, second, and third biological replicates related to Figure 4. For the upper left panel, biotinylated HKU1 RBD-loaded SA tips were dipped into 100 nM TMPRSS2 for 200 seconds followed by dissociation for 500 seconds. For all other replicates, biotinylated HKU1 isolate N1 RBD-loaded SA tips were dipped into 100 nM TMPRSS2 for 300 seconds followed by dissociation for 500 seconds. (D) QQ plot, homoscedasticity plot, and residual plot (calculated using Graphpad Prism 10) of the logarithmically scaled area under the BLI curve data shown in Figure 4. Consistent with a normal distribution, the QQ plot shows approximate linearity, while the homoscedasticity and residual plots show approximately random distributions.

FigS5 Figure S5, related to Figure 3. Enzymatic characterization of TMPRSS2 inhibition by the wildtype isolate N1 and alanine interface mutant HKU1 RBDs. (A) Raw data used for Figure 3B; the upper left panel shows the color key used for the other panels. Data show measured fluorescence (RFU) over time with 3.4 nM TMPRSS2 (harboring the N-terminal SUMO fusion, the C379-T447C disulfide bond and the N249 glycan) incubated with 0.19 mM Boc-QAR-AMC at 22°C in the presence of various concentrations of HKU1 isolate N1 RBD and alanine interface mutant RBDs. (B) Raw data and calculations used for Figure 3C–D; the upper left panel shows the color key used for the other panels. Lower left panels show a summary of AMC standard curves used to calculate AMC release from the Boc-QAR-AMC peptide substrate with 215, 72, and 24 nM HKU1 RBD, accounting for the inner filter effect. The middle panels show measured fluorescence (RFU) over time with 5 nM TMPRSS2 and 215, 72, or 24 nM HKU1 RBD (wildtype) or 215 nM HKU1 R517A RBD, incubated with various concentrations of Boc-QAR-AMC at 22 °C. The right panel shows the change in calculated AMC concentration over time.

FigS6 Figure S6, related to Figure 4. Evaluation of cell-surface expression of a panel of TMPRSS2 orthologs. **A**, Flow cytometry gating scheme. **B,** Cell surface expression of each transiently transfected TMPRSS2 ortholog determined by labeling HEK293T cells with an anti-flag tag antibody (α-FLAG-FITC) and measuring FITC fluorescence intensity by flow cytometry. The y-axis of each histogram is presented as a modal scale proportional to the maximum cell count for that plot (ranging between 3,500 and 8,000 singleton events for each ortholog).

TableS1

FigS1 Figure S1, related to Figure 1. Design and functional characterization of TMPRSS2 ectodomain constructs. (A) Reducing and non-reducing SDS-PAGE analysis of the samples shown in Figure 1B, including the intermediate step between enterokinase (EK) digestion and size-exclusion chromatography (SEC) purification. (B) Summary of AMC standard curves used to calculate AMC release from the Boc-QAR-AMC peptide substrate accounting for the inner filter effect. The left panel shows the relationship between AMC concentration and measured fluorescence (RFU). The lower inset shows the numerical equations of the linear relationships shown in the top panel. The right panel shows the calculated AMC concentration for each known AMC concentration shown in the left panel, using the equations in the lower inset at the indicated Boc-QAR-AMC concentrations. (C) Raw data and calculations used for Figure 1C; the color key is identical to Figure S1B. The left panels show measured fluorescence (RFU) over time for the indicated TMPRSS2 constructs at a concentration of 6.8 nM, whereas the right panel shows the change in calculated AMC concentration over time using the equations from Figure S1B. (D) Reducing SDS-PAGE used for analysis shown in Figure 1D. Expected products (i) and (ii), based on TMPRSS2 cleavage at the S_2_’ site, are labeled, along with additional cleavage products (asterisks). Reaction progress was monitored by densitometry of the S peak only.

FigS2 Figure S2, related to Figure 2. CryoEM data processing of the TMPRSS2-bound HKU1 RBD dataset (A-B) Representative electron micrograph and 2D class averages of the TMPRSS2-bound HKU1 RBD complex embedded in vitreous ice. The scale bars represent 100 nm and 150Å respectively. (C) Gold-standard Fourier shell correlation curve. The 0.143 cutoff is indicated by a horizontal dashed line. (D) Local resolution estimation calculated using cryoSPARC and plotted on the unsharpened (left) and sharpened (right) maps. (E) Data processing flowchart. CTF: contrast transfer function; NUR: non-uniform refinement. The angular distribution calculated in cryoSPARC for particle projections is shown. The heat map shows the number of particles for each viewing angle.

FigureS1

FigureS2

Data S1 Human TMPRSS2 SNPs at the HKU1 RBD-interacting site present in the GnomADv4 database.

Data S2 Human TMPRSS2 SNPs at the HKU1 RBD-interacting site present in the Regeneron Million Exome Variant database.

## Figures and Tables

**Table 1.** Cryo-EM data collection, refinement and validation statistics

	HKU1 RBD - TMPRSS2 PDB8VGT EMD-43224

**Data collection and processing**	
Magnification	105,000
Voltage (kV)	300
Electron exposure (e−/Å^2^)	60
Defocus range (μm)	−0.2 – −3.5
Pixel size (Å)	0.835
Symmetry imposed	C1
Initial particle images (no.)	8,831,644
Final particle images (no.)	810,357
Map resolution (Å)	2.9
FSC threshold	0.143
**Refinement**	
Model resolution (Å)	3.1
FSC threshold	0.5
Map sharpening *B* factor (Å^2^)	−130
Model composition	
Non-hydrogen atoms	4714
Protein residues	629
Ligands	5
*B* factors (Å^2^)	
Protein	15.84
Ligand	28.76
R.m.s. deviations	
Bond lengths (Å)	0.010
Bond angles (°)	1.107
**Validation**	
MolProbity score	1.27
Clashscore	2.03
Rotamer outliers (%)	0.93
Ramachandran plot	
Favored (%)	95.64
Allowed (%)	3.88
Disallowed (%)	0.48

**Key resources table:** 

REAGENT or RESOURCE	SOURCE	IDENTIFIER
Antibodies		
anti-VSV-G antibody	ATCC	Cat# CRL-2700
Monoclonal ANTI-FLAG^®^ M2 antibody produced in mouse	Sigma	F3165-1MG
Goat anti-Mouse IgG Fc Secondary Antibody, FITC	ThermoFisher Scientific	Cat# 31547
		
		
Bacterial and virus strains		
pcDNA3.1(+) HKU1_Spike_HAtag_S357A mutation without 355 glycation, YP_173238 (isolate N1, genotype: A)	This Study	n/a
pcDNA3.1(+) HKU1 Spike HAtag wt YP_173238 (isolate N1, genotype: A) N1 full length spike with C-terminal 19 residues deleted and add HA tag	This Study	n/a
Biological samples		
ExpiFectamine 293 Transfection Kit	ThermoFisher Scientific	Cat# A14525
ExpiFectamine CHO Transfection Kit	ThermoFisher Scientific	Cat# A29131
Biotin ligase (BirA) reaction kit	Avidity	Cat# 341113
SA biosensors	Sartorius	Cat# 18-5019
ONE-Glo^™^ EX Luciferase Assay System	Promega	Cat# E8150
293T/17 [HEK 293T/17]	ATCC	Cat# CRL-11268
		
Chemicals, peptides, and recombinant proteins		
Boc-Gln-Ala-Arg-AMC-HCl Fluorogenic Peptide	GLP BIO	GA20991
EKMax^™^ Enterokinase	ThermoFisher Scientific	E18001
Deposited data		
HKU1 RBD - TMPRSS2 complex CryoEM structure	https://www.ebi.ac.uk/pdbe/emdb/	EMD-43224, PDB 8VGT
Experimental models: Cell lines		
HEK293T cells	ATCC	Cat# CRL-11268
Expi293F cells	Thermo Fisher Scientific	Cat# A14527
ExpiCHO cells	ThermoFisher Scientific	Cat# A29127
Recombinant DNA		
pcDNA3.1(+): HKU1 isolate N1 Spike full length	This study	N/A
pcDNA3.1(+): HKU1 isolate N1 Spike RBD	This study	N/A
pcDNA3.1(+): HKU1 isolate N1 Spike RBD, (H488A, E505A, V509A, L510A, W515A, R517A or Y528A HKU1 RBD mutants)	This study	N/A
pCMV: SARS-CoV-2 S 2P (Wuhan-Hu-1) ectodomain	This study	N/A
pCMVR::TMPRSS2 ectodomain + S441A	This study	N/A
pCMVR::TMPRSS2 ectodomain + DS mutation	This study	N/A
pCMVR::TMPRSS2 ectodomain + DS mutation + S441A	This study	N/A
pCMVR::TMPRSS2 ectodomain + DS mutation	This study	N/A
pCMVR::SUMO-TMPRSS2 ectodomain + DS mutation + S441A	This study	N/A
pCMVR::SUMO-TMPRSS2 ectodomain + DS mutation	This study	N/A
pCMVR::SUMO-TMPRSS2 ectodomain + DS mutation + N249 glycan + S441A	This study	N/A
pCMVR::SUMO-TMPRSS2 ectodomain + DS mutation + N249 glycan (also with the following mutations: S412N, A468P, T341S, R413V, S412N+Y469L, R409K/T, R413K, V415I, K340D, L419M, Y414S/R/L, D417N, Y469N/L, L419A, S463T/F/Y, and R470K)	This study	N/A
pCMVR::SUMO-TMPRSS2 (from House Mouse) ectodomain + DS mutation + N249 glycan + S441A (also with S412N)		
pcDNA3.1(+)::full length TMPRSS2 + S441A with C-terminal 3X flag tag (human, chimpanzee, capuchin, marmoset, green monkey, house mouse, natal mouse, brown rat, hamster, mole rat, pika, camel, alpaca, goat, cow, flying fox, horseshoe bat, ferret, chicken, chameleon, and frog)		
pcDNA3.1(+)::full length TMPRSS2		
Software and algorithms		
cryoSPARC v4.4.0	(Punjani et al^102^)	https://cryosparc.com
Relion v3.0	(Zivanov et al^105^)	https://www3.mrc-lmb.cam.ac.uk/relion
Coot	(Emsley et al^109^)	https://www2.mrc-lmb.cam.ac.uk/personal/pemsley/coot/
Prism 10	GraphPad Software	https://www.graphpad.com/scientific-
Phenix	(Liebschner et al^113^)	https://www.phenix-online.org/download/
Chimera	(Pettersen et al^108^)	https://www.cgl.ucsf.edu/chimerax/
ProteinMPNN	(Dauparas et al^57^)	https://github.com/dauparas/ProteinMPNN
Excel	Microsoft	https://www.microsoft.com/en-us/
iTOL v6	(Letunic et al^92^)	https://itol.embl.de/
WebLogo	(Crooks et al^93^)	https://weblogo.berkeley.edu/logo.cgi
MEGA11	(Tamura et al^96^)	https://www.megasoftware.net/

## References

[1] Woo PCY, Lau SKP, Chu C-M, Chan K-H, Tsoi H-W, Huang Y, Wong BHL, Poon RWS, Cai JJ, Luk W-K, (2005). Characterization and complete genome sequence of a novel coronavirus, coronavirus HKU1, from patients with pneumonia. J. Virol. 79, 884–895. 15613317 10.1128/JVI.79.2.884-895.2005 PMC538593

[2] Góes LG, Durigon EL, Campos AA, Hein N, Passos SD, and Jerez JA (2011). Coronavirus HKU1 in Children, Brazil, 1995. Emerging Infectious Disease journal 17, 1147. 10.3201/eid1706.101381 PMC3358201 21749800

[3] Esper F, Weibel C, Ferguson D, Landry ML, and Kahn JS (2006). Coronavirus HKU1 infection in the United States. Emerg. Infect. Dis. 12, 775–779. 16704837 10.3201/eid1205.051316 PMC3374449

[4] Ruohola A, Waris M, Allander T, Ziegler T, Heikkinen T, and Ruuskanen O (2009). Viral etiology of common cold in children, Finland. Emerg. Infect. Dis. 15, 344–346. 19193292 10.3201/eid1502.081468 PMC2657644

[5] Woo PCY, Lau SKP, Yip CCY, Huang Y, Tsoi H-W, Chan K-H, and Yuen K-Y (2006). Comparative analysis of 22 coronavirus HKU1 genomes reveals a novel genotype and evidence of natural recombination in coronavirus HKU1. J. Virol. 80, 7136–7145. 16809319 10.1128/JVI.00509-06 PMC1489027

[6] Sloots TP, McErlean P, Speicher DJ, Arden KE, Nissen MD, and Mackay IM (2006). Evidence of human coronavirus HKU1 and human bocavirus in Australian children. J. Clin. Virol. 35, 99–102. 16257260 10.1016/j.jcv.2005.09.008 PMC7108338

[7] Zeng Z-Q, Chen D-H, Tan W-P, Qiu S-Y, Xu D, Liang H-X, Chen M-X, Li X, Lin Z-S, Liu W-K, (2018). Epidemiology and clinical characteristics of human coronaviruses OC43, 229E, NL63, and HKU1: a study of hospitalized children with acute respiratory tract infection in Guangzhou, China. Eur. J. Clin. Microbiol. Infect. Dis. 37, 363–369. 29214503 10.1007/s10096-017-3144-z PMC5780525

[8] Frutos AM, Balmaseda A, Vydiswaran N, Patel M, Ojeda S, Brouwer A, Tutino R, Cai S, Bakker K, Sanchez N, (2023). Burden and seasonality of primary and secondary symptomatic common cold coronavirus infections in Nicaraguan children. Influenza Other Respi. Viruses 17, e13078. 10.1111/irv.13086 PMC9835451 36494188

[9] Woo PCY, Lau SKP, Tsoi H-W, Huang Y, Poon RWS, Chu C-M, Lee RA, Luk W-K, Wong GKM, Wong BHL, (2005). Clinical and Molecular Epidemiological Features of Coronavirus HKU1–Associated Community-Acquired Pneumonia. J. Infect. Dis. 192, 1898–1907. 16267760 10.1086/497151 PMC7110183

[10] Tortorici MA, and Veesler D (2019). Structural insights into coronavirus entry. Adv. Virus Res. 105, 93–116. 31522710 10.1016/bs.aivir.2019.08.002 PMC7112261

[11] Li W, Moore MJ, Vasilieva N, Sui J, Wong SK, Berne MA, Somasundaran M, Sullivan JL, Luzuriaga K, Greenough TC, (2003). Angiotensin-converting enzyme 2 is a functional receptor for the SARS coronavirus. Nature 426, 450–454. 14647384 10.1038/nature02145 PMC7095016

[12] Walls AC, Park YJ, Tortorici MA, Wall A, McGuire AT, and Veesler D (2020). Structure, Function, and Antigenicity of the SARS-CoV-2 Spike Glycoprotein. Cell 181, 281–292.e6. 32155444 10.1016/j.cell.2020.02.058 PMC7102599

[13] Letko M, Marzi A, and Munster V (2020). Functional assessment of cell entry and receptor usage for SARS-CoV-2 and other lineage B betacoronaviruses. Nat Microbiol 5, 562–569. 32094589 10.1038/s41564-020-0688-y PMC7095430

[14] Zhou P, Yang XL, Wang XG, Hu B, Zhang L, Zhang W, Si HR, Zhu Y, Li B, Huang CL, (2020). A pneumonia outbreak associated with a new coronavirus of probable bat origin. Nature. 10.1038/s41586-020-2012-7. PMC7095418 32015507

[15] Hofmann H, Pyrc K, van der Hoek L, Geier M, Berkhout B, and Pohlmann S (2005). Human coronavirus NL63 employs the severe acute respiratory syndrome coronavirus receptor for cellular entry. Proc. Natl. Acad. Sci. U. S. A. 102, 7988–7993. 15897467 10.1073/pnas.0409465102 PMC1142358

[16] van der Hoek L, Pyrc K, Jebbink MF, Vermeulen-Oost W, Berkhout RJM, Wolthers KC, Wertheim-van Dillen PME, Kaandorp J, Spaargaren J, and Berkhout B (2004). Identification of a new human coronavirus. Nat. Med. 10, 368–373. 15034574 10.1038/nm1024 PMC7095789

[17] Xiong Q, Cao L, Ma C, Tortorici MA, Liu C, Si J, Liu P, Gu M, Walls AC, Wang C, (2022). Close relatives of MERS-CoV in bats use ACE2 as their functional receptors. Nature 612, 748–757. 36477529 10.1038/s41586-022-05513-3 PMC9734910

[18] Ma C, Liu C, Xiong Q, Yu X, Chen Y, Si J, Liu P, Tong F, Huang M, and Yan H (2023). Identification of ACE2 as the Entry Receptor for Two Novel European Bat Merbecoviruses. bioRxiv, 2023.10.02.560486. 10.1101/2023.10.02.560486.

[19] Letko M (2024). Functional assessment of cell entry and receptor use for merbecoviruses. bioRxiv, 2024.03.13.584892. 10.1101/2024.03.13.584892.

[20] Yeager CL, Ashmun RA, Williams RK, Cardellichio CB, Shapiro LH, Look AT, and Holmes KV (1992). Human aminopeptidase N is a receptor for human coronavirus 229E. Nature 357, 420–422. 1350662 10.1038/357420a0 PMC7095410

[21] Tortorici MA, Walls AC, Joshi A, Park Y-J, Eguia RT, Miranda MC, Kepl E, Dosey A, Stevens-Ayers T, Boeckh MJ, (2022). Structure, receptor recognition, and antigenicity of the human coronavirus CCoV-HuPn-2018 spike glycoprotein. Cell 185, 2279–2291.e17. 35700730 10.1016/j.cell.2022.05.019 PMC9135795

[22] Li W, Hulswit RJG, Kenney SP, Widjaja I, Jung K, Alhamo MA, van Dieren B, van Kuppeveld FJM, Saif LJ, and Bosch BJ (2018). Broad receptor engagement of an emerging global coronavirus may potentiate its diverse cross-species transmissibility. Proc. Natl. Acad. Sci. U. S. A. 115, E5135–E5143. 29760102 10.1073/pnas.1802879115 PMC5984533

[23] Delmas B, Gelfi J, L’Haridon R, Vogel Sjöström, Norén H, and Laude H (1992). Aminopeptidase N is a major receptor for the enteropathogenic coronavirus TGEV. Nature 357, 417–420. 1350661 10.1038/357417a0 PMC7095137

[24] Liu C, Tang J, Ma Y, Liang X, Yang Y, Peng G, Qi Q, Jiang S, Li J, Du L, (2015). Receptor usage and cell entry of porcine epidemic diarrhea coronavirus. J. Virol. 89, 6121–6125. 25787280 10.1128/JVI.00430-15 PMC4442452

[25] Raj VS, Mou H, Smits SL, Dekkers DHW, Müller MA, Dijkman R, Muth D, Demmers JAA, Zaki A, Fouchier RAM, (2013). Dipeptidyl peptidase 4 is a functional receptor for the emerging human coronavirus-EMC. Nature 495, 251–254. 23486063 10.1038/nature12005 PMC7095326

[26] Yang Y, Du L, Liu C, Wang L, Ma C, Tang J, Baric RS, Jiang S, and Li F (2014). Receptor usage and cell entry of bat coronavirus HKU4 provide insight into bat-to-human transmission of MERS coronavirus. Proc. Natl. Acad. Sci. U. S. A. 111, 12516–12521. 25114257 10.1073/pnas.1405889111 PMC4151778

[27] Wang Q, Qi J, Yuan Y, Xuan Y, Han P, Wan Y, Ji W, Li Y, Wu Y, Wang J, (2014). Bat origins of MERS-CoV supported by bat coronavirus HKU4 usage of human receptor CD26. Cell Host Microbe 16, 328–337. 25211075 10.1016/j.chom.2014.08.009 PMC7104937

[28] Chen J, Yang X, Si H, Gong Q, Que T, Li J, Li Y, Wu C, Zhang W, Chen Y, (2023). A bat MERS-like coronavirus circulates in pangolins and utilizes human DPP4 and host proteases for cell entry. Cell 186, 850–863.e16. 36803605 10.1016/j.cell.2023.01.019 PMC9933427

[29] Dveksler GS, Pensiero MN, Cardellichio CB, Williams RK, Jiang GS, Holmes KV, and Dieffenbach CW (1991). Cloning of the mouse hepatitis virus (MHV) receptor: expression in human and hamster cell lines confers susceptibility to MHV. J. Virol. 65, 6881–6891. 1719235 10.1128/jvi.65.12.6881-6891.1991 PMC250787

[30] Baggen J, Jacquemyn M, Persoons L, Vanstreels E, Pye VE, Wrobel AG, Calvaresi V, Martin SR, Roustan C, Cronin NB, (2023). TMEM106B is a receptor mediating ACE2-independent SARS-CoV-2 cell entry. Cell 186, 3427–3442.e22. 37421949 10.1016/j.cell.2023.06.005 PMC10409496

[31] Tortorici MA, Walls AC, Lang Y, Wang C, Li Z, Koerhuis D, Boons GJ, Bosch BJ, Rey FA, de Groot RJ, (2019). Structural basis for human coronavirus attachment to sialic acid receptors. Nat. Struct. Mol. Biol. 26, 481–489. 31160783 10.1038/s41594-019-0233-y PMC6554059

[32] Hulswit RJG, Lang Y, Bakkers MJG, Li W, Li Z, Schouten A, Ophorst B, van Kuppeveld FJM, Boons GJ, Bosch BJ, (2019). Human coronaviruses OC43 and HKU1 bind to 9-O-acetylated sialic acids via a conserved receptor-binding site in spike protein domain A. Proc. Natl. Acad. Sci. U. S. A. 10.1073/pnas.1809667116. PMC6377473 30679277

[33] Vlasak R, Luytjes W, Spaan W, and Palese P (1988). Human and bovine coronaviruses recognize sialic acid-containing receptors similar to those of influenza C viruses. Proc. Natl. Acad. Sci. U. S. A. 85, 4526–4529. 3380803 10.1073/pnas.85.12.4526 PMC280463

[34] Li Z, Lang Y, Liu L, Bunyatov MI, Sarmiento AI, de Groot RJ, and Boons G-J (2021). Synthetic O-acetylated sialosides facilitate functional receptor identification for human respiratory viruses. Nat. Chem. 13, 496–503. 33753916 10.1038/s41557-021-00655-9

[35] Hurdiss DL, Drulyte I, Lang Y, Shamorkina TM, Pronker MF, van Kuppeveld FJM, Snijder J, and de Groot RJ (2020). Cryo-EM structure of coronavirus-HKU1 haemagglutinin esterase reveals architectural changes arising from prolonged circulation in humans. Nat. Commun. 11, 4646. 32938911 10.1038/s41467-020-18440-6 PMC7495468

[36] Bakkers MJ, Lang Y, Feitsma LJ, Hulswit RJ, de Poot SA, van Vliet AL, Margine I, de Groot-Mijnes JD, van Kuppeveld FJ, Langereis MA, (2017). Betacoronavirus Adaptation to Humans Involved Progressive Loss of Hemagglutinin-Esterase Lectin Activity. Cell Host Microbe 21, 356–366. 28279346 10.1016/j.chom.2017.02.008 PMC7104930

[37] Saunders N, Fernandez I, Planchais C, Michel V, Rajah MM, Baquero Salazar E, Postal J, Porrot F, Guivel-Benhassine F, Blanc C, (2023). TMPRSS2 is a functional receptor for human coronavirus HKU1. Nature. 10.1038/s41586-023-06761-7. PMC11331971 37879362

[38] Pronker MF, Creutznacher R, Drulyte I, Hulswit RJG, Li Z, van Kuppeveld FJM, Snijder J, Lang Y, Bosch B-J, Boons G-J, (2023). Sialoglycan binding triggers spike opening in a human coronavirus. Nature. 10.1038/s41586-023-06599-z. PMC10700143 37794193

[39] Tomlins SA, Rhodes DR, Perner S, Dhanasekaran SM, Mehra R, Sun X-W, Varambally S, Cao X, Tchinda J, Kuefer R, (2005). Recurrent fusion of TMPRSS2 and ETS transcription factor genes in prostate cancer. Science 310, 644–648. 16254181 10.1126/science.1117679

[40] Hoffmann M, Kleine-Weber H, Schroeder S, Krüger N, Herrler T, Erichsen S, Schiergens TS, Herrler G, Wu NH, Nitsche A, (2020). SARS-CoV-2 Cell Entry Depends on ACE2 and TMPRSS2 and Is Blocked by a Clinically Proven Protease Inhibitor. Cell 181, 271–280.e8. 32142651 10.1016/j.cell.2020.02.052 PMC7102627

[41] Zang R, Gomez Castro MF, McCune BT, Zeng Q, Rothlauf PW, Sonnek NM, Liu Z, Brulois KF, Wang X, Greenberg HB, (2020). TMPRSS2 and TMPRSS4 promote SARS-CoV-2 infection of human small intestinal enterocytes. Sci Immunol 5. 10.1126/sciimmunol.abc3582. PMC7285829 32404436

[42] Glowacka I, Bertram S, Müller MA, Allen P, Soilleux E, Pfefferle S, Steffen I, Tsegaye TS, He Y, Gnirss K, (2011). Evidence that TMPRSS2 activates the severe acute respiratory syndrome coronavirus spike protein for membrane fusion and reduces viral control by the humoral immune response. J. Virol. 85, 4122–4134. 21325420 10.1128/JVI.02232-10 PMC3126222

[43] Heurich A, Hofmann-Winkler H, Gierer S, Liepold T, Jahn O, and Pöhlmann S (2014). TMPRSS2 and ADAM17 cleave ACE2 differentially and only proteolysis by TMPRSS2 augments entry driven by the severe acute respiratory syndrome coronavirus spike protein. J. Virol. 88, 1293–1307. 24227843 10.1128/JVI.02202-13 PMC3911672

[44] Shirato K, Kawase M, and Matsuyama S (2013). Middle East respiratory syndrome coronavirus infection mediated by the transmembrane serine protease TMPRSS2. J. Virol. 87, 12552–12561. 24027332 10.1128/JVI.01890-13 PMC3838146

[45] Gierer S, Bertram S, Kaup F, Wrensch F, Heurich A, Krämer-Kühl A, Welsch K, Winkler M, Meyer B, Drosten C, (2013). The spike protein of the emerging betacoronavirus EMC uses a novel coronavirus receptor for entry, can be activated by TMPRSS2, and is targeted by neutralizing antibodies. J. Virol. 87, 5502–5511. 23468491 10.1128/JVI.00128-13 PMC3648152

[46] Bertram S, Dijkman R, Habjan M, Heurich A, Gierer S, Glowacka I, Welsch K, Winkler M, Schneider H, Hofmann-Winkler H, (2013). TMPRSS2 activates the human coronavirus 229E for cathepsin-independent host cell entry and is expressed in viral target cells in the respiratory epithelium. J. Virol. 87, 6150–6160. 23536651 10.1128/JVI.03372-12 PMC3648130

[47] Limburg H, Harbig A, Bestle D, Stein DA, Moulton HM, Jaeger J, Janga H, Hardes K, Koepke J, Schulte L, (2019). TMPRSS2 Is the Major Activating Protease of Influenza A Virus in Primary Human Airway Cells and Influenza B Virus in Human Type II Pneumocytes. J. Virol. 93. 10.1128/JVI.00649-19. PMC6803253 31391268

[48] Böttcher E, Matrosovich T, Beyerle M, Klenk H-D, Garten W, and Matrosovich M (2006). Proteolytic activation of influenza viruses by serine proteases TMPRSS2 and HAT from human airway epithelium. J. Virol. 80, 9896–9898. 16973594 10.1128/JVI.01118-06 PMC1617224

[49] Shapira T, Monreal IA, Dion SP, Buchholz DW, Imbiakha B, Olmstead AD, Jager M, Désilets A, Gao G, Martins M, (2022). A TMPRSS2 inhibitor acts as a pan-SARS-CoV-2 prophylactic and therapeutic. Nature 605, 340–348. 35344983 10.1038/s41586-022-04661-w PMC9095466

[50] Hamilton BS, Chung C, Cyphers SY, Rinaldi VD, Marcano VC, and Whittaker GR (2014). Inhibition of influenza virus infection and hemagglutinin cleavage by the protease inhibitor HAI-2. Biochem. Biophys. Res. Commun. 450, 1070–1075. 24978308 10.1016/j.bbrc.2014.06.109 PMC4465281

[51] Mahoney M, Damalanka VC, Tartell MA, Chung DH, Lourenço AL, Pwee D, Mayer Bridwell AE, Hoffmann M, Voss J, Karmakar P, (2021). A novel class of TMPRSS2 inhibitors potently block SARS-CoV-2 and MERS-CoV viral entry and protect human epithelial lung cells. Proc. Natl. Acad. Sci. U. S. A. 118. 10.1073/pnas.2108728118. PMC8694051 34635581

[52] Fraser BJ, Beldar S, Seitova A, Hutchinson A, Mannar D, Li Y, Kwon D, Tan R, Wilson RP, Leopold K, (2022). Structure and activity of human TMPRSS2 protease implicated in SARS-CoV-2 activation. Nat. Chem. Biol. 18, 963–971. 35676539 10.1038/s41589-022-01059-7

[53] Walls AC, Tortorici MA, Frenz B, Snijder J, Li W, Rey FA, DiMaio F, Bosch B-J, and Veesler D (2016). Glycan shield and epitope masking of a coronavirus spike protein observed by cryo-electron microscopy. Nat. Struct. Mol. Biol. 23, 899–905. 27617430 10.1038/nsmb.3293 PMC5515730

[54] Walls AC, Xiong X, Park YJ, Tortorici MA, Snijder J, Quispe J, Cameroni E, Gopal R, Dai M, Lanzavecchia A, (2019). Unexpected Receptor Functional Mimicry Elucidates Activation of Coronavirus Fusion. Cell 176, 1026–1039.e15. 30712865 10.1016/j.cell.2018.12.028 PMC6751136

[55] Ou X, Guan H, Qin B, Mu Z, Wojdyla JA, Wang M, Dominguez SR, Qian Z, and Cui S (2017). Crystal structure of the receptor binding domain of the spike glycoprotein of human betacoronavirus HKU1. Nat. Commun. 8, 15216. 28534504 10.1038/ncomms15216 PMC5529671

[56] Herter S, Piper DE, Aaron W, Gabriele T, Cutler G, Cao P, Bhatt AS, Choe Y, Craik CS, Walker N, (2005). Hepatocyte growth factor is a preferred in vitro substrate for human hepsin, a membrane-anchored serine protease implicated in prostate and ovarian cancers. Biochem. J 390, 125–136. 15839837 10.1042/BJ20041955 PMC1184568

[57] Dauparas J, Anishchenko I, Bennett N, Bai H, Ragotte RJ, Milles LF, Wicky BIM, Courbet A, de Haas RJ, Bethel N, (2022). Robust deep learning–based protein sequence design using ProteinMPNN. Science 378, 49–56. 36108050 10.1126/science.add2187 PMC9997061

[58] Vernon RM, Chong PA, Tsang B, Kim TH, Bah A, Farber P, Lin H, and Forman-Kay JD (2018). Pi-Pi contacts are an overlooked protein feature relevant to phase separation. Elife 7. 10.7554/eLife.31486. PMC5847340 29424691

[59] Walls AC, Tortorici MA, Snijder J, Xiong X, Bosch BJ, Rey FA, and Veesler D (2017). Tectonic conformational changes of a coronavirus spike glycoprotein promote membrane fusion. Proc. Natl. Acad. Sci. U. S. A. 114, 11157–11162. 29073020 10.1073/pnas.1708727114 PMC5651768

[60] Walls AC, Tortorici MA, Bosch BJ, Frenz B, Rottier PJM, DiMaio F, Rey FA, and Veesler D (2016). Cryo-electron microscopy structure of a coronavirus spike glycoprotein trimer. Nature 531, 114–117. 26855426 10.1038/nature16988 PMC5018210

[61] Tortorici MA, Beltramello M, Lempp FA, Pinto D, Dang HV, Rosen LE, McCallum M, Bowen J, Minola A, Jaconi S, (2020). Ultrapotent human antibodies protect against SARS-CoV-2 challenge via multiple mechanisms. Science 370, 950–957. 32972994 10.1126/science.abe3354 PMC7857395

[62] Piccoli L, Park YJ, Tortorici MA, Czudnochowski N, Walls AC, Beltramello M, Silacci-Fregni C, Pinto D, Rosen LE, Bowen JE, (2020). Mapping Neutralizing and Immunodominant Sites on the SARS-CoV-2 Spike Receptor-Binding Domain by Structure-Guided High-Resolution Serology. Cell 183, 1024–1042.e21. 32991844 10.1016/j.cell.2020.09.037 PMC7494283

[63] Park Y-J, De Marco A, Starr TN, Liu Z, Pinto D, Walls AC, Zatta F, Zepeda SK, Bowen JE, Sprouse KR, (2022). Antibody-mediated broad sarbecovirus neutralization through ACE2 molecular mimicry. Science, eabm8143. 10.1126/science.abm8143 PMC9400459 34990214

[64] Corti D, Zhao J, Pedotti M, Simonelli L, Agnihothram S, Fett C, Fernandez-Rodriguez B, Foglierini M, Agatic G, Vanzetta F, (2015). Prophylactic and postexposure efficacy of a potent human monoclonal antibody against MERS coronavirus. Proc. Natl. Acad. Sci. U. S. A. 112, 10473–10478. 26216974 10.1073/pnas.1510199112 PMC4547275

[65] Traggiai E, Becker S, Subbarao K, Kolesnikova L, Uematsu Y, Gismondo MR, Murphy BR, Rappuoli R, and Lanzavecchia A (2004). An efficient method to make human monoclonal antibodies from memory B cells: potent neutralization of SARS coronavirus. Nat. Med. 10, 871–875. 15247913 10.1038/nm1080 PMC7095806

[66] Park Y-J, Pinto D, Walls AC, Liu Z, De Marco A, Benigni F, Zatta F, Silacci-Fregni C, Bassi J, Sprouse KR, (2022). Imprinted antibody responses against SARS-CoV-2 Omicron sublineages. Science, eadc9127. 10.1126/science.adc9127 PMC12945441 36264829

[67] Starr TN, Czudnochowski N, Liu Z, Zatta F, Park Y-J, Addetia A, Pinto D, Beltramello M, Hernandez P, Greaney AJ, (2021). SARS-CoV-2 RBD antibodies that maximize breadth and resistance to escape. Nature. 10.1038/s41586-021-03807-6. PMC9282883 34261126

[68] Starr TN, Zepeda SK, Walls AC, Greaney AJ, Alkhovsky S, Veesler D, and Bloom JD (2022). ACE2 binding is an ancestral and evolvable trait of sarbecoviruses. Nature 603, 913–918. 35114688 10.1038/s41586-022-04464-z PMC8967715

[69] Lee J, Zepeda SK, Park Y-J, Taylor AL, Quispe J, Stewart C, Leaf EM, Treichel C, Corti D, King NP, (2023). Broad receptor tropism and immunogenicity of a clade 3 sarbecovirus. Cell Host Microbe. 10.1016/j.chom.2023.10.018. PMC10913562 37989312

[70] Edridge AWD, Kaczorowska JM, Hoste ACR, Bakker M, Klein M, Jebbink MF, Matser A, Kinsella C, Rueda P, Prins M, (2020). Coronavirus protective immunity is short-lasting. medRxiv, 2020.05.11.20086439. 10.1038/s41591-020-1083-1 32929268

[71] Eguia RT, Crawford KHD, Stevens-Ayers T, Kelnhofer-Millevolte L, Greninger AL, Englund JA, Boeckh MJ, and Bloom JD (2021). A human coronavirus evolves antigenically to escape antibody immunity. PLoS Pathog. 17, e1009453. 33831132 10.1371/journal.ppat.1009453 PMC8031418

[72] Starr TN, Greaney AJ, Hilton SK, Ellis D, Crawford KHD, Dingens AS, Navarro MJ, Bowen JE, Tortorici MA, Walls AC, (2020). Deep Mutational Scanning of SARS-CoV-2 Receptor Binding Domain Reveals Constraints on Folding and ACE2 Binding. Cell 182, 1295–1310.e20. 32841599 10.1016/j.cell.2020.08.012 PMC7418704

[73] McCallum M, Czudnochowski N, Rosen LE, Zepeda SK, Bowen JE, Walls AC, Hauser K, Joshi A, Stewart C, Dillen JR, (2022). Structural basis of SARS-CoV-2 Omicron immune evasion and receptor engagement. Science, eabn8652. 35076256 10.1126/science.abn8652 PMC9427005

[74] Wong AHM, Tomlinson ACA, Zhou D, Satkunarajah M, Chen K, Sharon C, Desforges M, Talbot PJ, and Rini JM (2017). Receptor-binding loops in alphacoronavirus adaptation and evolution. Nat. Commun. 8, 1735. 29170370 10.1038/s41467-017-01706-x PMC5701055

[75] Wang C, Hesketh EL, Shamorkina TM, Li W, Franken PJ, Drabek D, van Haperen R, Townend S, van Kuppeveld FJM, Grosveld F, (2022). Antigenic structure of the human coronavirus OC43 spike reveals exposed and occluded neutralizing epitopes. Nat. Commun. 13, 2921. 35614127 10.1038/s41467-022-30658-0 PMC9132891

[76] Huang X, Dong W, Milewska A, Golda A, Qi Y, Zhu QK, Marasco WA, Baric RS, Sims AC, Pyrc K, (2015). Human Coronavirus HKU1 Spike Protein Uses O-Acetylated Sialic Acid as an Attachment Receptor Determinant and Employs Hemagglutinin-Esterase Protein as a Receptor-Destroying Enzyme. J. Virol. 89, 7202–7213. 25926653 10.1128/JVI.00854-15 PMC4473545

[77] Widjaja I, Wang C, van Haperen R, Gutierrez-Alvarez J, van Dieren B, Okba NMA, Raj VS, Li W, Fernandez-Delgado R, Grosveld F, (2019). Towards a solution to MERS: protective human monoclonal antibodies targeting different domains and functions of the MERS-coronavirus spike glycoprotein. Emerg. Microbes Infect. 8, 516–530. 30938227 10.1080/22221751.2019.1597644 PMC6455120

[78] Yuan Y, Cao D, Zhang Y, Ma J, Qi J, Wang Q, Lu G, Wu Y, Yan J, Shi Y, (2017). Cryo-EM structures of MERS-CoV and SARS-CoV spike glycoproteins reveal the dynamic receptor binding domains. Nat. Commun. 8, 15092. 28393837 10.1038/ncomms15092 PMC5394239

[79] Pallesen J, Wang N, Corbett KS, Wrapp D, Kirchdoerfer RN, Turner HL, Cottrell CA, Becker MM, Wang L, Shi W, (2017). Immunogenicity and structures of a rationally designed prefusion MERS-CoV spike antigen. Proc. Natl. Acad. Sci. U. S. A. 114, E7348–E7357. 28807998 10.1073/pnas.1707304114 PMC5584442

[80] Kirchdoerfer RN, Cottrell CA, Wang N, Pallesen J, Yassine HM, Turner HL, Corbett KS, Graham BS, McLellan JS, and Ward AB (2016). Pre-fusion structure of a human coronavirus spike protein. Nature 531, 118–121. 26935699 10.1038/nature17200 PMC4860016

[81] Xiong X, Tortorici MA, Snijder J, Yoshioka C, Walls AC, Li W, McGuire AT, Rey FA, Bosch BJ, and Veesler D (2018). Glycan Shield and Fusion Activation of a Deltacoronavirus Spike Glycoprotein Fine-Tuned for Enteric Infections. J. Virol. 92. 10.1128/JVI.01628-17. PMC5790929 29093093

[82] Shang J, Zheng Y, Yang Y, Liu C, Geng Q, Tai W, Du L, Zhou Y, Zhang W, and Li F (2018). Cryo-electron microscopy structure of porcine Deltacoronavirus spike protein in the prefusion state. J. Virol. 92. 10.1128/JVI.01556-17. PMC5790952 29070693

[83] Shang J, Zheng Y, Yang Y, Liu C, Geng Q, Luo C, Zhang W, and Li F (2018). Cryo-EM structure of infectious bronchitis coronavirus spike protein reveals structural and functional evolution of coronavirus spike proteins. PLoS Pathog. 14, e1007009. 29684066 10.1371/journal.ppat.1007009 PMC5933801

[84] Li Z, Tomlinson AC, Wong AH, Zhou D, Desforges M, Talbot PJ, Benlekbir S, Rubinstein JL, and Rini JM (2019). The human coronavirus HCoV-229E S-protein structure and receptor binding. Elife 8. 10.7554/eLife.51230. PMC6970540 31650956

[85] Lau SKP, Woo PCY, Li KSM, Tsang AKL, Fan RYY, Luk HKH, Cai J-P, Chan K-H, Zheng B-J, Wang M, (2015). Discovery of a novel coronavirus, China Rattus coronavirus HKU24, from Norway rats supports the murine origin of Betacoronavirus 1 and has implications for the ancestor of Betacoronavirus lineage A. J. Virol. 89, 3076–3092. 25552712 10.1128/JVI.02420-14 PMC4337523

[86] Addetia A, Piccoli L, Case JB, Park Y-J, Beltramello M, Guarino B, Dang H, de Melo GD, Pinto D, Sprouse K, (2023). Neutralization, effector function and immune imprinting of Omicron variants. Nature 621, 592–601. 37648855 10.1038/s41586-023-06487-6 PMC10511321

[87] McCallum M, De Marco A, Lempp FA, Tortorici MA, Pinto D, Walls AC, Beltramello M, Chen A, Liu Z, Zatta F, (2021). N-terminal domain antigenic mapping reveals a site of vulnerability for SARS-CoV-2. Cell 184, 2332–2347.e16. 33761326 10.1016/j.cell.2021.03.028 PMC7962585

[88] Carrion R Jr, Ro Y, Hoosien K, Ticer A, Brasky K, de la Garza M, Mansfield K, and Patterson JL (2011). A small nonhuman primate model for filovirus-induced disease. Virology 420, 117–124. 21959017 10.1016/j.virol.2011.08.022 PMC3195836

[89] Chiu CY, Sánchez-San Martín C, Bouquet J, Li T, Yagi S, Tamhankar M, Hodara VL, Parodi LM, Somasekar S, Yu G, (2017). Experimental Zika Virus Inoculation in a New World Monkey Model Reproduces Key Features of the Human Infection. Sci. Rep. 7, 17126. 29215081 10.1038/s41598-017-17067-w PMC5719425

[90] Boyer JD, Ugen KE, Wang B, Agadjanyan M, Gilbert L, Bagarazzi ML, Chattergoon M, Frost P, Javadian A, Williams WV, (1997). Protection of chimpanzees from high-dose heterologous HIV-1 challenge by DNA vaccination. Nat. Med. 3, 526–532. 9142121 10.1038/nm0597-526

[91] Girard M, Kieny MP, Pinter A, Barre-Sinoussi F, Nara P, Kolbe H, Kusumi K, Chaput A, Reinhart T, and Muchmore E (1991). Immunization of chimpanzees confers protection against challenge with human immunodeficiency virus. Proc. Natl. Acad. Sci. U. S. A. 88, 542–546. 1988952 10.1073/pnas.88.2.542 PMC50847

[92] Letunic I, and Bork P (2021). Interactive Tree Of Life (iTOL) v5: an online tool for phylogenetic tree display and annotation. Nucleic Acids Res. 49, W293–W296. 33885785 10.1093/nar/gkab301 PMC8265157

[93] Crooks GE, Hon G, Chandonia J-M, and Brenner SE (2004). WebLogo: a sequence logo generator. Genome Res. 14, 1188–1190. 15173120 10.1101/gr.849004 PMC419797

[94] Sechan F, Grobben M, Edridge AWD, Jebbink MF, Loens K, Ieven M, Goossens H, van Hemert-Glaubitz S, van Gils MJ, and van der Hoek L (2022). Atypical Antibody Dynamics During Human Coronavirus HKU1 Infections. Front. Microbiol. 13, 853410. 35572703 10.3389/fmicb.2022.853410 PMC9093712

[95] Ieven M, Coenen S, Loens K, Lammens C, Coenjaerts F, Vanderstraeten A, Henriques-Normark B, Crook D, Huygen K, Butler CC, (2018). Aetiology of lower respiratory tract infection in adults in primary care: a prospective study in 11 European countries. Clin. Microbiol. Infect. 24, 1158–1163. 29447989 10.1016/j.cmi.2018.02.004 PMC7129248

[96] Tamura K, Stecher G, and Kumar S (2021). MEGA11: Molecular Evolutionary Genetics Analysis Version 11. Mol. Biol. Evol. 38, 3022–3027. 33892491 10.1093/molbev/msab120 PMC8233496

[97] Cheng J, Novati G, Pan J, Bycroft C, Žemgulytė A, Applebaum T, Pritzel A, Wong LH, Zielinski M, Sargeant T, (2023). Accurate proteome-wide missense variant effect prediction with AlphaMissense. Science 381, eadg7492. 37733863 10.1126/science.adg7492

[98] Russo CJ, and Passmore LA (2014). Electron microscopy: Ultrastable gold substrates for electron cryomicroscopy. Science 346, 1377–1380. 25504723 10.1126/science.1259530 PMC4296556

[99] Tan YZ, Baldwin PR, Davis JH, Williamson JR, Potter CS, Carragher B, and Lyumkis D (2017). Addressing preferred specimen orientation in single-particle cryo-EM through tilting. Nat. Methods 14, 793–796. 28671674 10.1038/nmeth.4347 PMC5533649

[100] Suloway C, Pulokas J, Fellmann D, Cheng A, Guerra F, Quispe J, Stagg S, Potter CS, and Carragher B (2005). Automated molecular microscopy: the new Leginon system. J. Struct. Biol. 151, 41–60. 15890530 10.1016/j.jsb.2005.03.010

[101] Tegunov D, and Cramer P (2019). Real-time cryo-electron microscopy data preprocessing with Warp. Nat. Methods 16, 1146–1152. 31591575 10.1038/s41592-019-0580-y PMC6858868

[102] Punjani A, Rubinstein JL, Fleet DJ, and Brubaker MA (2017). cryoSPARC: algorithms for rapid unsupervised cryo-EM structure determination. Nat. Methods 14, 290–296. 28165473 10.1038/nmeth.4169

[103] Bepler T, Morin A, Rapp M, Brasch J, Shapiro L, Noble AJ, and Berger B (2019). Positive-unlabeled convolutional neural networks for particle picking in cryo-electron micrographs. Nat. Methods 16, 1153–1160. 31591578 10.1038/s41592-019-0575-8 PMC6858545

[104] Punjani A, Zhang H, and Fleet DJ (2020). Non-uniform refinement: adaptive regularization improves single-particle cryo-EM reconstruction. Nat. Methods 17, 1214–1221. 33257830 10.1038/s41592-020-00990-8

[105] Zivanov J, Nakane T, Forsberg BO, Kimanius D, Hagen WJ, Lindahl E, and Scheres SH (2018). New tools for automated high-resolution cryo-EM structure determination in RELION-3. Elife 7. 10.7554/eLife.42166. PMC6250425 30412051

[106] Rosenthal PB, and Henderson R (2003). Optimal determination of particle orientation, absolute hand, and contrast loss in single-particle electron cryomicroscopy. J. Mol. Biol. 333, 721–745. 14568533 10.1016/j.jmb.2003.07.013

[107] Chen S, McMullan G, Faruqi AR, Murshudov GN, Short JM, Scheres SH, and Henderson R (2013). High-resolution noise substitution to measure overfitting and validate resolution in 3D structure determination by single particle electron cryomicroscopy. Ultramicroscopy 135, 24–35. 23872039 10.1016/j.ultramic.2013.06.004 PMC3834153

[108] Pettersen EF, Goddard TD, Huang CC, Couch GS, Greenblatt DM, Meng EC, and Ferrin TE (2004). UCSF Chimera--a visualization system for exploratory research and analysis. J. Comput. Chem. 25, 1605–1612. 15264254 10.1002/jcc.20084

[109] Emsley P, Lohkamp B, Scott WG, and Cowtan K (2010). Features and development of Coot. Acta Crystallogr. D Biol. Crystallogr. 66, 486–501. 20383002 10.1107/S0907444910007493 PMC2852313

[110] Frenz B, Rämisch S, Borst AJ, Walls AC, Adolf-Bryfogle J, Schief WR, Veesler D, and DiMaio F (2019). Automatically Fixing Errors in Glycoprotein Structures with Rosetta. Structure 27, 134–139.e3. 30344107 10.1016/j.str.2018.09.006 PMC6616339

[111] Wang RY, Song Y, Barad BA, Cheng Y, Fraser JS, and DiMaio F (2016). Automated structure refinement of macromolecular assemblies from cryo-EM maps using Rosetta. Elife 5. 10.7554/eLife.17219. PMC5115868 27669148

[112] Chen VB, Arendall WB, Headd JJ, Keedy DA, Immormino RM, Kapral GJ, Murray LW, Richardson JS, and Richardson DC (2010). MolProbity: all-atom structure validation for macromolecular crystallography. Acta Crystallogr. D Biol. Crystallogr. 66, 12–21. 20057044 10.1107/S0907444909042073 PMC2803126

[113] Liebschner D, Afonine PV, Baker ML, Bunkóczi G, Chen VB, Croll TI, Hintze B, Hung LW, Jain S, McCoy AJ, (2019). Macromolecular structure determination using X-rays, neutrons and electrons: recent developments in Phenix. Acta Crystallogr D Struct Biol 75, 861–877. 31588918 10.1107/S2059798319011471 PMC6778852

[114] Agirre J, Iglesias-Fernández J, Rovira C, Davies GJ, Wilson KS, and Cowtan KD (2015). Privateer: software for the conformational validation of carbohydrate structures. Nat. Struct. Mol. Biol. 22, 833–834. 26581513 10.1038/nsmb.3115

