# Supplementary figures and images for: Human coronavirus HKU1 recognition of the TMPRSS2 host receptor
Source: Cell. Author manuscript; Available in PMC 2026 Jan 29. (PMC12854727; doi:10.1016/j.cell.2024.06.006)

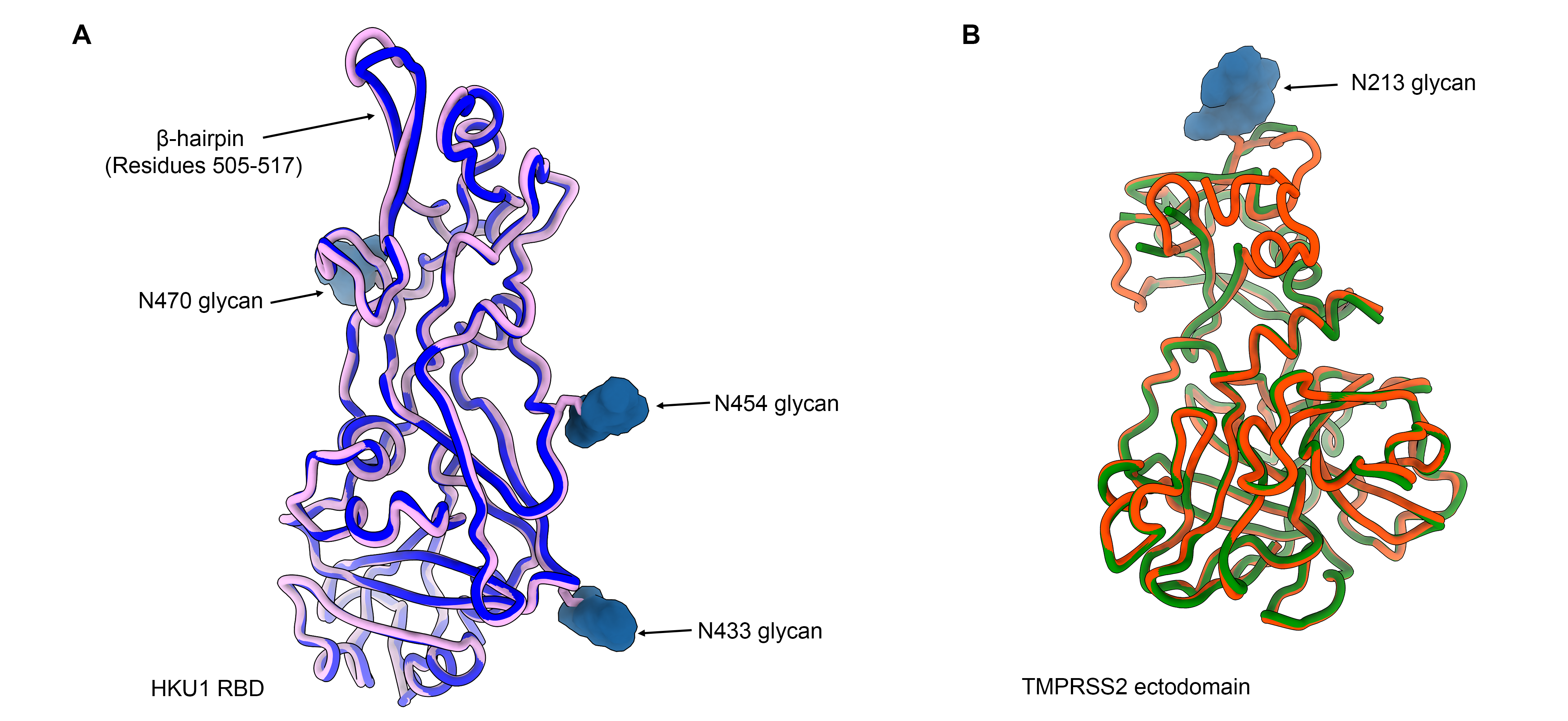

Supplement: FigS3 — (A) Ribbon diagram of the cryoEM structure of the HKU1 RBD (purple) bound to the human TMPRSS2 ectodomain superimposed to the crystal structure of the apo HKU1 RBD (blue, PDB 5KWB). The TMPRSS2 ectodomain is omitted for clarity. (B) Ribbon diagram of the cryoEM structure of the human TMPRSS2 ectodomain (orange) bound to the HKU1 RBD superimposed to the crystal structure of the nafamostat-bound TMPRSS2 (green, PDB 7MEQ). The HKU1 RBD is omitted for clarity. [file NIHMS2094799-supplement-FigS3.tif]

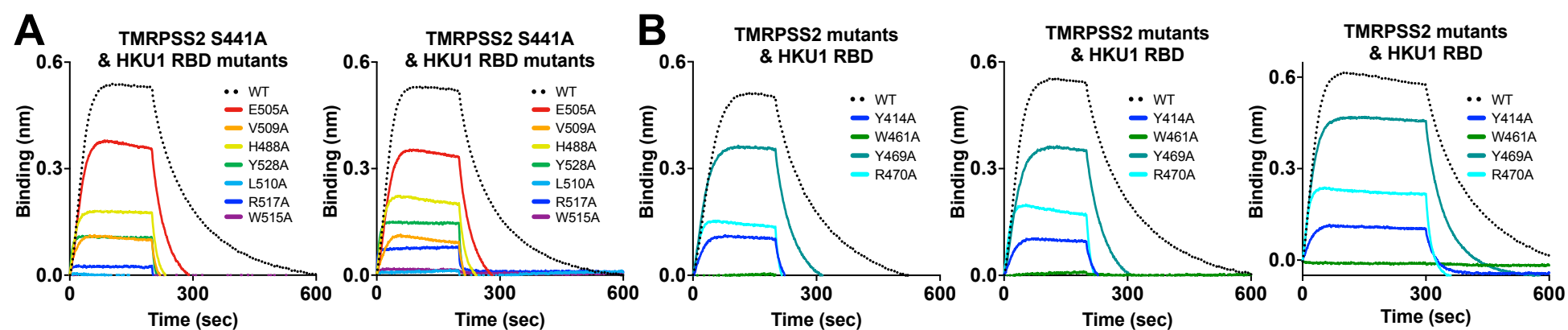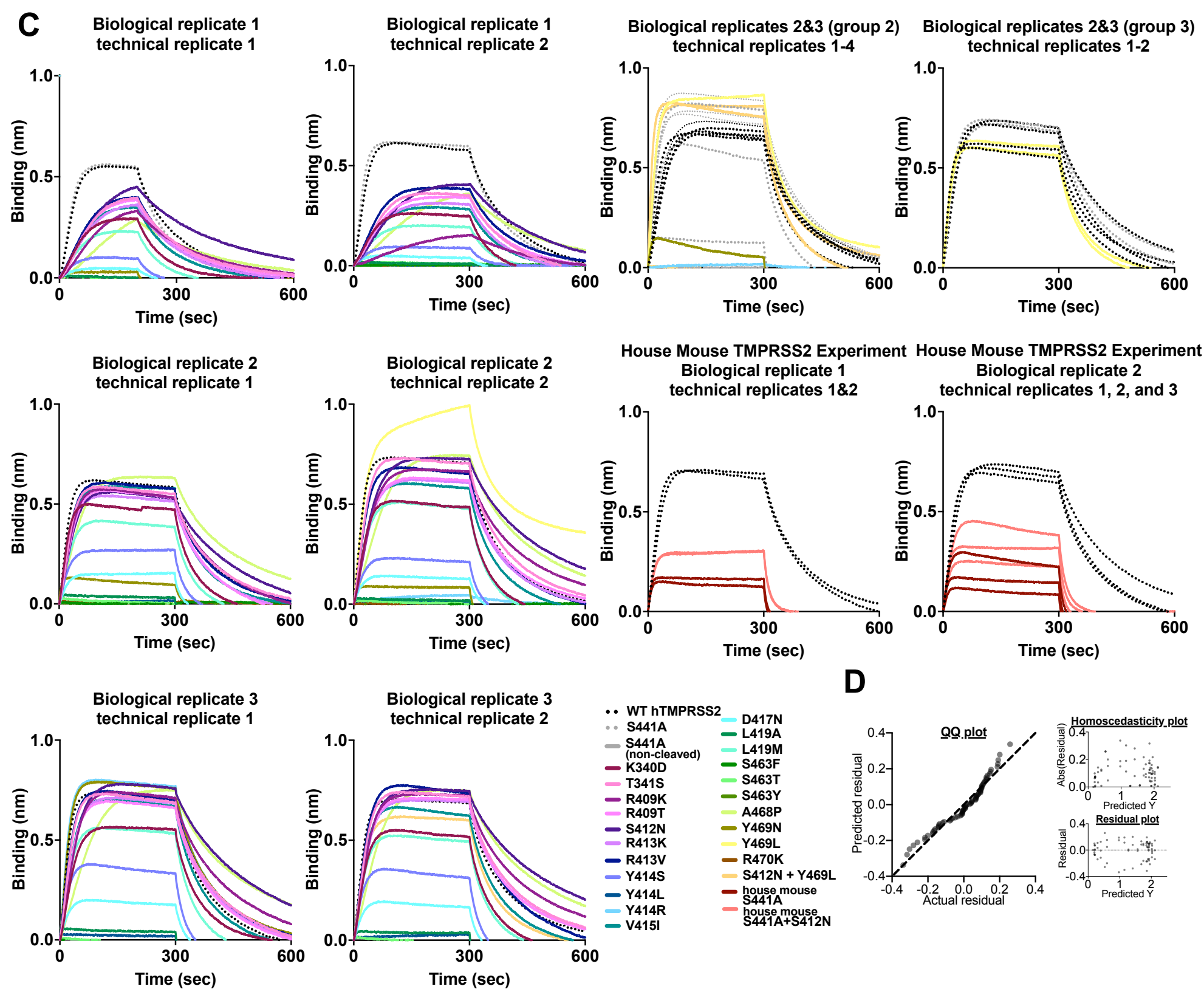

Supplement: FigS4 — (A) Two panels show baseline-subtracted response curves for the first (left panel) and second (right panel) biological replicates of TMPRSS2 S441A binding to HKU1 RBD mutants (related to Figure 2). Biotinylated HKU1 isolate N1 RBD-loaded SA tips were dipped into 100 nM TMPRSS2 S441A for 200 seconds followed by dissociation for 400 seconds. (B) Three panels show baseline-subtracted response curves for the first, second, and third biological replicates of TMPRSS2 mutants binding to the HKU1 RBD (related to Figure 2). For the first and second biological replicate (first two panels), biotinylated HKU1 isolate N1 RBD-loaded SA tips were dipped into 100 nM TMPRSS2 mutants for 200 seconds followed by dissociation for 400 seconds. For the third biological replicate (last panel), biotinylated HKU1 isolate N1 RBD-loaded SA tips were dipped into 100 nM TMPRSS2 mutants for 300 seconds followed by dissociation for 500 seconds. (C) Baseline-subtracted response curves for the first, second, and third biological replicates related to Figure 4. For the upper left panel, biotinylated HKU1 RBD-loaded SA tips were dipped into 100 nM TMPRSS2 for 200 seconds followed by dissociation for 500 seconds. For all other replicates, biotinylated HKU1 isolate N1 RBD-loaded SA tips were dipped into 100 nM TMPRSS2 for 300 seconds followed by dissociation for 500 seconds. (D) QQ plot, homoscedasticity plot, and residual plot (calculated using Graphpad Prism 10) of the logarithmically scaled area under the BLI curve data shown in Figure 4. Consistent with a normal distribution, the QQ plot shows approximate linearity, while the homoscedasticity and residual plots show approximately random distributions. [file NIHMS2094799-supplement-FigS4.pdf]

**A**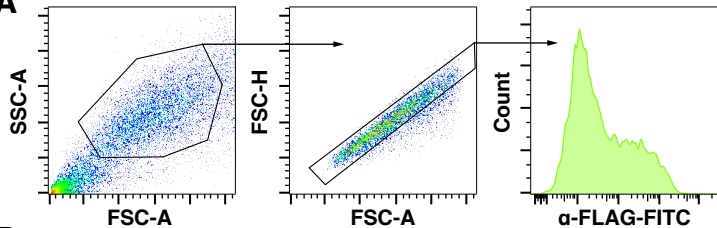**B**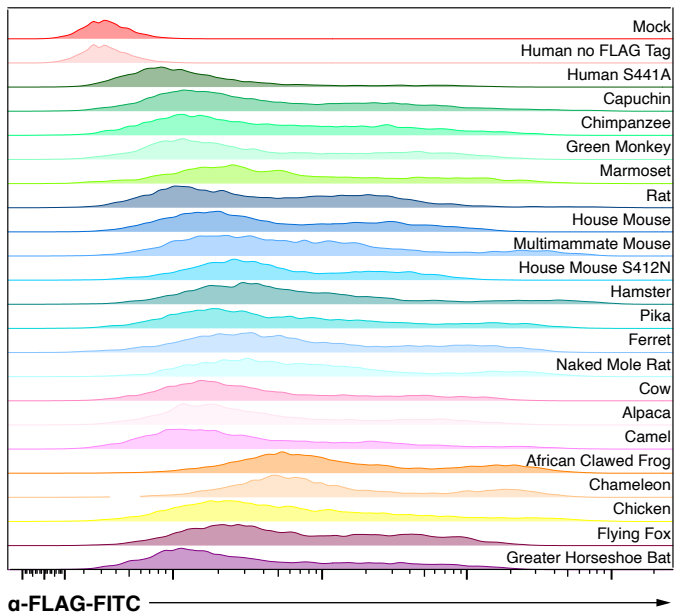

Supplement: FigS6 — A, Flow cytometry gating scheme. B, Cell surface expression of each transiently transfected TMPRSS2 ortholog determined by labeling HEK293T cells with an anti-flag tag antibody (α-FLAG-FITC) and measuring FITC fluorescence intensity by flow cytometry. The y-axis of each histogram is presented as a modal scale proportional to the maximum cell count for that plot (ranging between 3,500 and 8,000 singleton events for each ortholog). [file NIHMS2094799-supplement-FigS6.pdf]

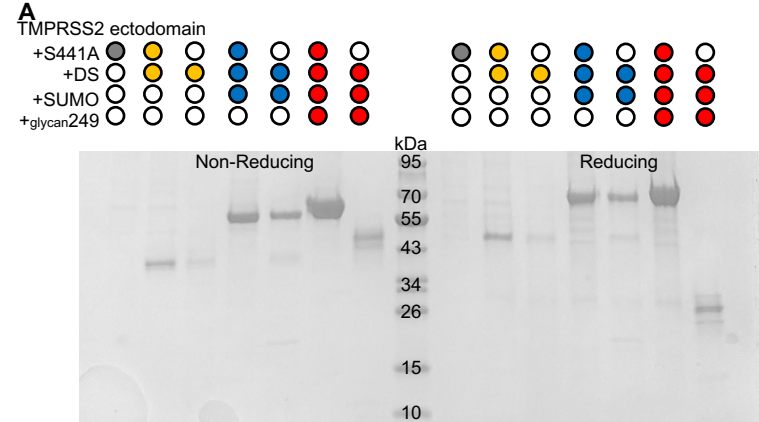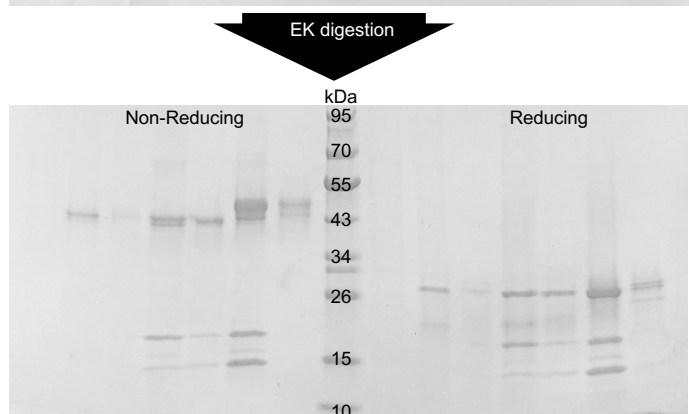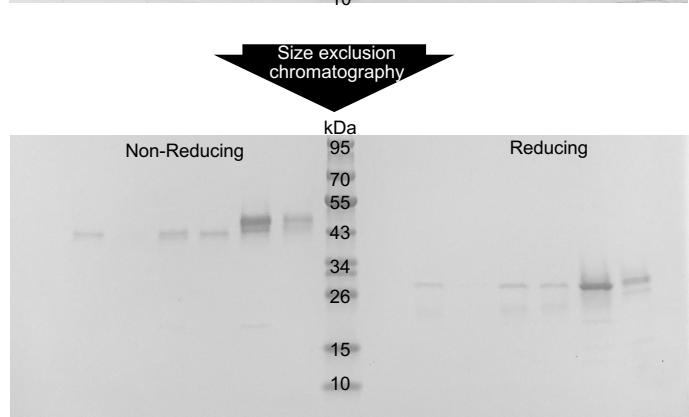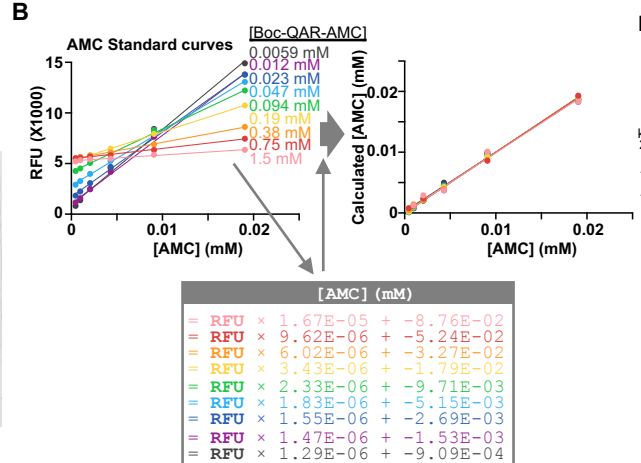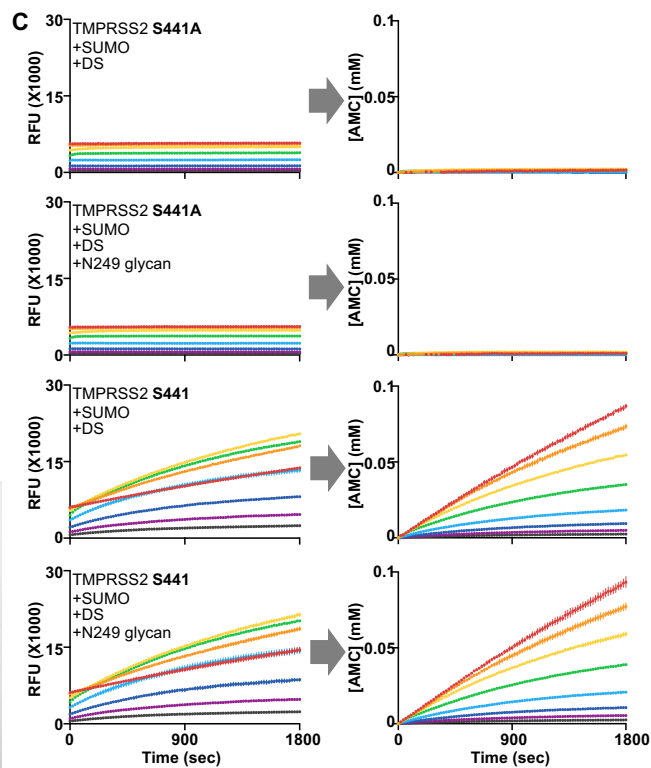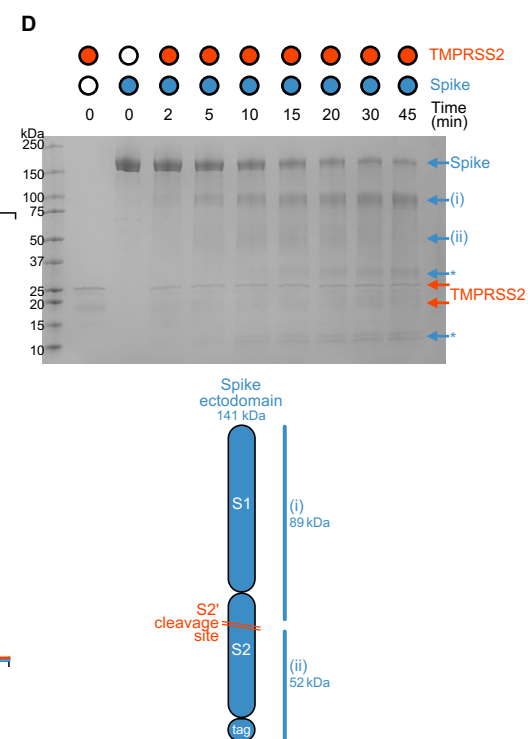

Supplement: FigS1 — (A) Reducing and non-reducing SDS-PAGE analysis of the samples shown in Figure 1B, including the intermediate step between enterokinase (EK) digestion and size-exclusion chromatography (SEC) purification. (B) Summary of AMC standard curves used to calculate AMC release from the Boc-QAR-AMC peptide substrate accounting for the inner filter effect. The left panel shows the relationship between AMC concentration and measured fluorescence (RFU). The lower inset shows the numerical equations of the linear relationships shown in the top panel. The right panel shows the calculated AMC concentration for each known AMC concentration shown in the left panel, using the equations in the lower inset at the indicated Boc-QAR-AMC concentrations. (C) Raw data and calculations used for Figure 1C; the color key is identical to Figure S1B. The left panels show measured fluorescence (RFU) over time for the indicated TMPRSS2 constructs at a concentration of 6.8 nM, whereas the right panel shows the change in calculated AMC concentration over time using the equations from Figure S1B. (D) Reducing SDS-PAGE used for analysis shown in Figure 1D. Expected products (i) and (ii), based on TMPRSS2 cleavage at the S2’ site, are labeled, along with additional cleavage products (asterisks). Reaction progress was monitored by densitometry of the S peak only. [file NIHMS2094799-supplement-FigS1.pdf]

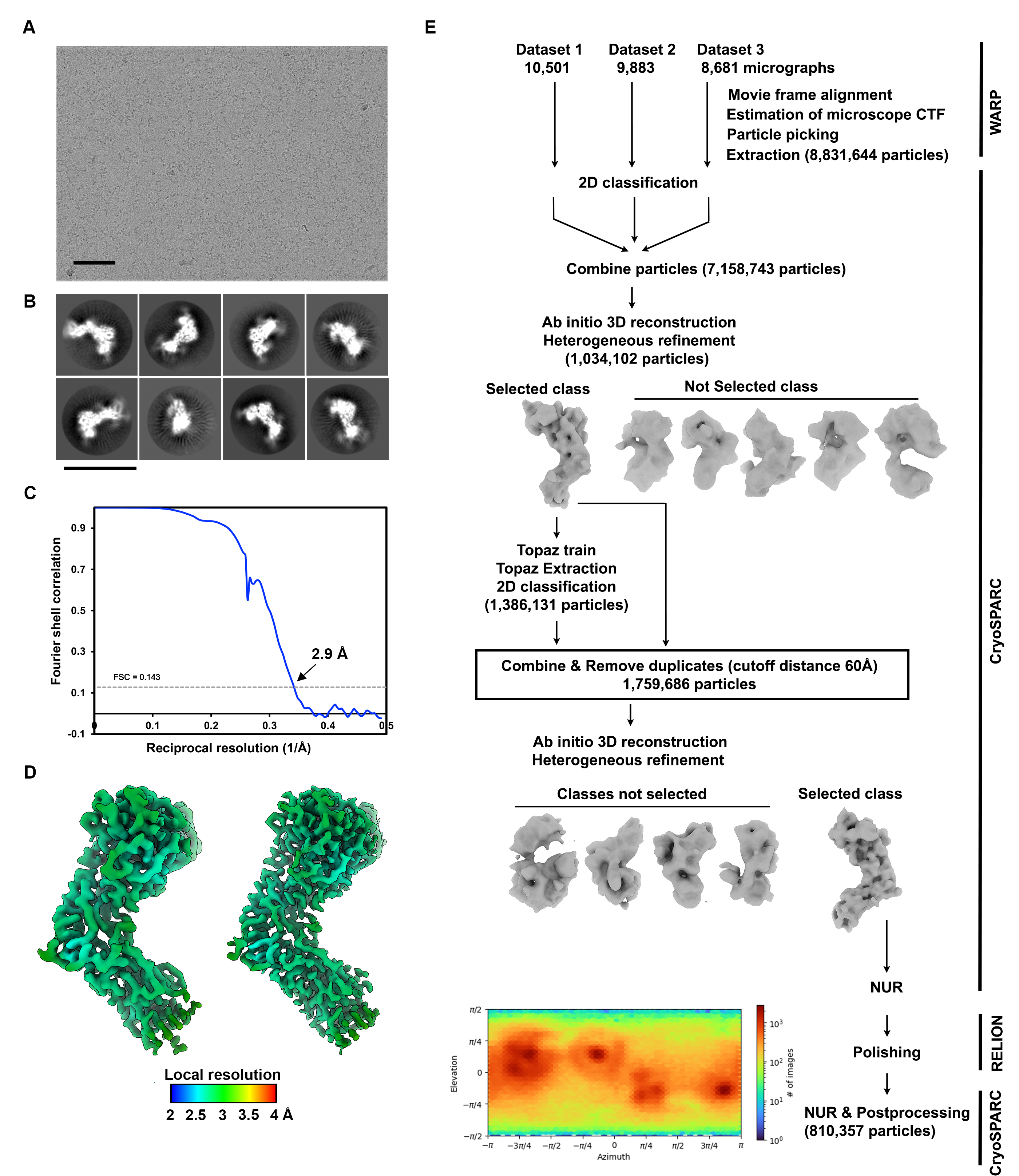

Supplement: FigS2 — (A-B) Representative electron micrograph and 2D class averages of the TMPRSS2-bound HKU1 RBD complex embedded in vitreous ice. The scale bars represent 100 nm and 150Å respectively. (C) Gold-standard Fourier shell correlation curve. The 0.143 cutoff is indicated by a horizontal dashed line. (D) Local resolution estimation calculated using cryoSPARC and plotted on the unsharpened (left) and sharpened (right) maps. (E) Data processing flowchart. CTF: contrast transfer function; NUR: non-uniform refinement. The angular distribution calculated in cryoSPARC for particle projections is shown. The heat map shows the number of particles for each viewing angle. [file NIHMS2094799-supplement-FigS2.tif]
