# Supplementary material for: Human coronavirus HKU1 recognition of the TMPRSS2 host receptor
Source: Cell. Author manuscript; Available in PMC 2026 Jan 29. (PMC12854727; doi:10.1016/j.cell.2024.06.006)
Supplement: TableS1 [file NIHMS2094799-supplement-TableS1.pdf]

Full length TMPRSS2 (S441A) Mus musculus Q3UKE3 C-term 3XFLAG:
MALNSGSPPGIGPCYENHGYQSEHICPPRPPVAPNGYNLYPAQYYPSPVPQYAPRITTQASTSVIHTHPKSSGALCTSKSKKSLCLALALGTVLTGAAVAAVLLWR
FWDSNCSTSEMECGSSGTCISSSLWCDGVAHCPNGEDENRCVRLYGQSFILQVYSSQRKAWYPVCQDDWSESYGRAACKDMGYKNNFYSSQGIPDQSGATS
FMKLNVSSGNVDLYKKLYHSDSCSSRMVVSLRCIECGVRSVKRQSRIVGGLNASPGDWPWQVSLHVQGVHVCGGSIITPEWIVTAAHCVEEPLSSPRYWTAFAG
ILRQSLMFYGSRHQVEKVISHPNYDSKTKNNDIALMKLQTPLAFNDLVKPVCLPSPGMMLDLDQECWISGWGATYEKGKTSDVLNAAMVPLIEPSKCNSKYIYNNL
ITPAMICAGFLQGSVDSCQGDSGGPLVTLKNGIWWLIGDTSWGSGCAKALRPGVYGNVTVFTDWIYKQMRANSSGDYKDHDGDYKDHDIDYKDDDDK

Full length TMPRSS2 (S441A) Ratus Norvegicus Q6P7D7 C-term 3XFLAG:
MALNSGSPPGIGPYYENHGYQSEHVYSPRPPVSPSGYNLYPAQSCPSPVPQYAPRVTTQASTPAIHIQPRSSGTLCTSKSKKSMLVALALGTVLAGAAVAAGLLW
KFWDSKCSSSEMECGSSGTCISSSLWCDGVSQCPNGEDENRCVRLYGTSFTLQVYSSQRKAWYPVCQDDWNESYGRAACKDMGYKNSFYSSQGIPDQSGAT
SFMKLNVSAGNVDLYKKLYHSDSCSSRMVVSLRCIECGVRSVRRQSRIVGGSTASPGDWPWQVSLHVQGIHVCGGSIITPEWIVTAAHCVEEPLSSPRYWTAFA
GILKQSLMFYGSRHQVEKVISHPNYDSKTKNNDIALMKLQTPLAFNDVVKPVCLPNPGMMLDLAQECWISGWGATYEKGKTSDVLNAAMVPLIEPSKCNSKYIYN
NLITPAMICAGFLQGSVDSCQGDSGGPLVTLKNEIWWLIGDTSWGSGCAKAYRPGVYGNVTVFTDWIYQQMRANSSGDYKDHDGDYKDHDIDYKDDDDK

Full length TMPRSS2 (S441A) Rhinolophus ferrumequinum A0A671DXW4 C-term 3XFLAG:
MALNSGSPPGVGPYYENHGYQPESLYPPRPAVAPSAYSVYPARYYTPVVPQYTPRVMTHTSTPAIHTQPKSPSGTLCTSKTKKALCITFSLGAVLVGAVVAAVLL
WKFMENKCSVSGIECGSSGTCVSASHWCDGILHCPSGEDENRCVRLYGPNFILQVYSSQRKSWHPVCQDDWSENYGRAACQDMGYRNSFYSSQGIVDDSGA
TSFMKLNISAGHTDLYKKLYHSDVCSSKTVVSLRCIECGVNTKMSRQSRIVGGTSAALGDWPWQVSLHVQGVHVCGGSIITPEWIVTAAHCVEEPLNNPRYWTAF
AGILRQSFMFYGSGYRVEKVISHPNYDSKSKNNDIALMKLQTPLTFNDKVKPVCLPNPGLMLEPTQSCWISGWGATYEKGKTSEVLNAVMVPLIEPWQCNSKYVY
NNLITPAMICAGYLQGTIDSCQGDSGGPLVTLKSSVWWLIGDTSWGSGCAKANRPGVYGNVTVFTDWIYRQMRANSSGDYKDHDGDYKDHDIDYKDDDDK

Full length TMPRSS2 (S441A) Chlorocebus sabaeusgreen A0A0D9R7A1 C-term 3XFLAG:
MALNSGSPPGVGPYYENHGYQPENPYPAQPTVAPNVYEVHPAQYYPSPVPQYIPSVLTHASNPAVRTQPKSPSGTVCTSKTKKALCVTMTLGAVLVGAALAAGL
LWKFMGSKCSDSGIECDSSGTCISSSNWCDGVSHCPSGEDENRCVRLYGPNFILQVYSSQRKSWHPVCRDDWNENYARAACRDMGYQNRFYSSQGIADNSG
ATSFMKLNTSAGNVDIYKKLYHSDACSSKAVVSLRCIACGVRSNLSRQSRIVGGQNALPGAWPWQVSLHVQNIHVCGGSIITPEWIVTAAHCVEKPLNSPWQWTA
FAGILTQSSMFYVKGHRVEKVISHPNYDSKTKNNDIALMKLHTPLTFNELVKPVCLPNPGMMLEPEQHCWISGWGATQEKGKTSDMLNAAMVPLIEPRRCNSKRV
YDGLVTPAMICAGFLQGTVDSCQGDSGGPLVTLKNDVWWLIGDTSWGSGCAQANRPGVYGNVTVFTDWIYRQMRADDSGDYKDHDGDYKDHDIDYKDDDDK

Full length TMPRSS2 (S441A) Mesocricetus auratus hamster A0A1U8C7X1 C-term 3XFLAG:
MALNSGSPPGIGPYYENHGFQSEHIYPPRPPVAPDVYNPYPPQNYPPPVPQYFPRVTTQASTTVTHTQPHSSGKLCTSTSKTKKSLCFALSLGIVLVGAAVAAVLL
WKFLPGCSTSEMECMSSGTCISSSLWCDGTSHCPNGEDENRCVRLYGPSFTLQVYSSQRKAWYPVCQDDWNDSYGRAACKDMGYKNNFYYTQGIPDSSGAT
SFMKLNISAGNIDLYKKLYHSDSCSSRMVVSLRCIQCGVRSATRQSRIVGGSNASPGDWPWQVSLHVQGVHVCGGSIITPEWIVTAAHCVEEPLNSPRYWTAFAG
ILSQSLMFYGSRHQVEKVISHPNYNSETKNNDIALMKLQTPLTFNDLVKPVCLPNPGMMLDPAQECWISGWGSTYEKGKTSDMLNAAMVPLIERSKCNNKYIYNN
LITPAMVCAGFLQGTVDSCQGDSGGPLVTLKNDIWWLIGDTSWGSGCAKALRPGVYGNVTVFTDWIYQQMMANSSGDYKDHDGDYKDHDIDYKDDDDK

Full length TMPRSS2 (S441A) Mustela furo ferret A0A8U0SMZ2 C-term 3XFLAG:
MALNAGSPPGVGPYYENHGYQPESLYPAPPATVPSVYVAYPAAYYPPAVPQYTPRVLTQASTPAVRTQPKSPSGTACTAKAKKALCITISLGAVLAGAAVTAVLL
WKFMENKCSVSGIECGSSGTCISPSHWCDGVLHCPSGEDENRCVRLYGPNFILQVYSAQRKSWHPVCQDDWSDSYGRAACQDMGYRNSFYSSQGIVDDSGA
SSFMKLNTSAGNTDLYKKLYHSDICASKTVVSLRCIECGVAGKTMRQSRIVGGSSASPGDWPWQVSLHVQGTHVCGGSIITPEWIVTAAHCVEEPLNNPRYWTVF
AGVLRQSFMFYGHGYRVGKVISHPSYDSKTKNNDIALMKLQTPLTFSDKVKPVCLPNPGMMLEPNQSCWISGWGATHEKGKTSDELNAVMVPLIEPWRCNSKYV
YNSLVTPAMICAGYLRGGTDSCQGDSGGPLVTLKSRIWWLIGDTSWGSGCAKANRPGVYGNVTVFTDWIYRQMRANSSGDYKDHDGDYKDHDIDYKDDDDK

Full length TMPRSS2 (S441A) Camelus dromedarius XP_010993167.1 C-term 3XFLAG:
MALNSGSPPGVGPYYENHGYQPESFYPLKPSAASSAYMVYPAQYYPPAVPQYTPRVQTHTSTPVIRMQPKPPSETVCTSKTKKVLCVTLGLGAILVGAVLAAVLL
WKFKESRCSASEMECGSSGTCISPSQWCDGVLHCPGGEDENQCVRLYGPNFILQVYSPQRKSWHPVCQEDWSESFGRAMCQDLGYGNSFYSSQGVVDDSG
ATSFMKLNISANNIDLYKKLYHSDVCSSKRVVSLRCIECGVSEKTSRQSRIVGGSSANLGDWPWQVSLHVQGIHVCGGSIITPEWIVTAAHCVEQPLSNAKIWTAFA
GILSQSLMIYGNGYQIAKVISHPNYDSKTKNNDIALMKLQTPLTFNDRVKPVCLPNPGMMLEATQSCWISGWGATYEKGKTSDVLNAVMVHLIEPWKCNSKYVYN
NLITSAMICAGYLQGGVDSCQGDSGGPLVTLKNSVWWLLGDTSWGSGCAKAYRPGVYGNMTVFTDWIYRQMRANSSGDYKDHDGDYKDHDIDYKDDDDK

Full length TMPRSS2 (S441A) Gallus domesticus XP_015156666.1 C-term 3XFLAG:
MTSTVNPPPYYENHGFQTENYYSARPQVGANPYPQYFSTNVPSVPTYIPRVSTHQSSIPVAPPSSSSRMCSSSIKKIVIITLSILLVICCAIAAFLIWYFVENRCLGSLI
ECGSSGVCISPSVWCDGVTDCPNGEDENRCVRLYGPNFILEVYSPVSQTWYPVCQDDWTDDFGKIACEDMGYNVDTYYYSQGVAAEVSFKSFMKLNTSAGNT
DLYKRLQSSDYCASGNVVSLRCIECGLPTKSTAVMSRIVGGSMASLGQWPWQVSLHVQDTHVCGGSIITREWLVTAAHCVEGLFSDPYIWSVYAGILSQNEMHS
RPGYRVQKIISHPNYDTDSKDNDVALMKLETPLSFTNTIRPVCLPNPGMMFQPNQQCWISGWGAEYQGGKTANDLNYVMVPLIERSTCNSVYVYDGMVLPTMVC
AGYLQGGIDSCQGDSGGPLVTNKNSVWWLVGDTSWGTGCASPNRPGVYGNMTVFTDWIYKNMQANRSGDYKDHDGDYKDHDIDYKDDDDK

Full length TMPRSS2 (S441A) Chamaeleo jacksonii G1KE28 C-term 3XFLAG:
MNSRPPPYYENYAYQPENIAPPRHAGGYMYPPYPSPYYPSSVPHYIPRVSTNQSTPVPTVVQPKATPAKCMPKTRKAVCLFLAISVLLIGGAIAAVLIWHFVTDSCF
GSKIKCGTTGMCVAPSQWCDGIRDCPNNEDETRCVRLFGPEFQLEVYSSESKDWYPVCSDDWNDKHGKTACEDLGYNSNTYFKSQTIPLMSASKGFMKLNASA
GDIDLYKKLYNSKSCSSRSLVSLQCIDCGTRRVNRRNRIVGGTSASLGDWPWQVSLHSSGTHLCGGSIITPEWIVTAAHCVEKAFSNPNYWTVFAGILTQPEMISS
KGHKVAKVIPHPGYDTSSKTNDVALMKLQSPLVFDEFVRPVCLPNPGMMFQSDQPYWISGWGAVEQKGPTSKKLNAARIRLIDSDTCNNRYIYNGLILPTMICAGY
LNGGIDSCQGDSGGPLVTSKDSLWWLVGDTSWGTGCATKYRPGVYGNMTVFTDWIYKNMQANRRSGDYKDHDGDYKDHDIDYKDDDDK

Full length TMPRSS2 (S441A) Xenopus laevis XP_018104413.1 C-term 3XFLAG:
MAGRDYRWKSPSPPYFENYGFQQDNNNAFSRPVQPNFYEAFQPPRPQLSPAPHYIPQVSTIHSVPAINEHKSQTWWTPRRKKIACIVAATSVLIALLIVGAVLCWY
FVTMSCQMKCGTSGSCVRSIQWCDGVAQCPGGEDESYCVRMYGPDFQLQAYIPATSSWLSVCNENWGDSQGRSVCQDMGYSTYVKSSSVSATSATEGYLKL
NTSVVNGKLQSRIYKSSFCTSGVVTLRCIECGSSTKNVENRIVGGSQASLGDWPWQVSLQYNERHVCGGSIITSNYILTAAHCVEGAYSSPYAWTVYVGSISRSTA
GIRYYVKSVIGHQKYDTKTKNNDVALMRLKISILFSSTVQPVCLPNAGMPWASGQSCWTSGWGATYEGGTSSNVLNAAMVPLIDADTCNRPAVYNGAVTSTMICA
GYLRGGIDSCQGDSGGPLVTKTNSLWWLVGDTSWGTGCANVNKPGVYGNITEFLPWIFLQMQTYGSGDYKDHDGDYKDHDIDYKDDDDK

Full length TMPRSS2 (S441) Homo sapiens:
MALNSGSPPAIGPYYENHGYQPENPYPAQPTVVPTVYEVHPAQYYPSPVPQYAPRVLTQASNPVVCTQPKSPSGTVCTSKTKKALCITLTLGTFLVGAALAAGLL
WKFMGSKCSNSGIECDSSGTCINPSNWCDGVSHCPGGEDENRCVRLYGPNFILQVYSSQRKSWHPVCQDDWNENYGRAACRDMGYKNNFYSSQGIVDDSGS
TSFMKLNTSAGNVDIYKKLYHSDACSSKAVVSLRCIACGVNLNSSRQSRIVGGESALPGAWPWQVSLHVQNVHVCGGSIITPEWIVTAAHCVEKPLNNPWHWTAF
AGILRQSFMFYGAGYQVEKVISHPNYDSKTKNNDIALMKLQKPLTFNDLVKPVCLPNPGMMLQPEQLCWISGWGATEEKGKTSEVLNAAKVLLIETQRCNSRYVY
DNLITPAMICAGFLQGNVDSCQGDSGGPLVTSKNNIWWLIGDTSWGSGCAKAYRPGVYGNVMVFTDWIYRQMRADG

Full length TMPRSS2 (S441A) Homo sapiens C-term 3XFLAG:
MALNSGSPPAIGPYYENHGYQPENPYPAQPTVVPTVYEVHPAQYYPSPVPQYAPRVLTQASNPVVCTQPKSPSGTVCTSKTKKALCITLTLGTFLVGAALAAGLL
WKFMGSKCSNSGIECDSSGTCINPSNWCDGVSHCPGGEDENRCVRLYGPNFILQVYSSQRKSWHPVCQDDWNENYGRAACRDMGYKNNFYSSQGIVDDSGS
TSFMKLNTSAGNVDIYKKLYHSDACSSKAVVSLRCIACGVNLNSSRQSRIVGGESALPGAWPWQVSLHVQNVHVCGGSIITPEWIVTAAHCVEKPLNNPWHWTAF
AGILRQSFMFYGAGYQVEKVISHPNYDSKTKNNDIALMKLQKPLTFNDLVKPVCLPNPGMMLQPEQLCWISGWGATEEKGKTSEVLNAAKVLLIETQRCNSRYVY
DNLITPAMICAGFLQGNVDSCQGDAGGPLVTSKNNIWWLIGDTSWGSGCAKAYRPGVYGNVMVFTDWIYRQMRADGSGDYKDHDGDYKDHDIDYKDDDDK

Full length TMPRSS2 (S441A) TMPRSS2 pika XP_058517588.1 C-term 3XFLAG:
MAVNSGPAPGVGPYYENHGYQSEDLYARPPPVAPQAYAEYPAQYYPPTVPQYAPQVTTQATTPVVLRRSSSRLLCSAKVVLAVGAVLVGAGLIAGLLWNFLENRC
SGIECGSSGICISPSNWCDGVFHCPNGEDENRCVRLYGPNFILQVYSSQRNSWYPVCQDGWNENYGRAACQDMGYKSSFYSSDGIADDSGATSFMKLNLSASTG
DLYQKLYHSETCSSKVVVSLRCIRCGVKSSPSGQSRIVGGTSAVAGEWPWQVSLHVQGVHVCGGSIITPQWIVTAAHCLEEPLNSARYWTAYAGILSQPKMLYGSG
NRAEKVISHPDYDSKTKDNDIALIKLQTPLTFSEAIQPVCLPNPGLMLEPNQECWISGWGATYEKGKTSDVLNAAQVPLIESWKCNSRYVYNNLITPSMVCAGYLEGN
VDSCQGDAGGPLVTKKNSIWWLIGDTSWGSGCAQAYRPGVYGNVTAFTDWIYQQMRANSGGDYKDHDGDYKDHDIDYKDDDDK

Full length TMPRSS2 (S441A) chimp A0A6D2WBC5 C-term 3XFLAG:
MALNSGSPTAIGPYYENHGYQPENPYPAQPTVAPTVYEVHPAQYYPSPVPQYAPRVLTQASNPVVRMQPKSPSGTVCTSKTKKALCLTLTLGTFLVGAALAAGLLW
NFMGSKCSNSGIECDSSGTCISPSNWCDGVSHCPSGEDENRCVRLYGPNFILQVYSSQRKSWHPVCQDDWNENYGRAACRDMGYKNNFYSSQGIVDDSGSTSF
MKLNTSAGNVDIYKKLYHSDACSSKAVVSLRCIACGVNLNSSRQSRIVGGESALPGAWPWQVSLHVQNVHVCGGSIITPEWIVTAAHCVEKPLNNPWHWTAFAGIL
RQSFMFYGAGYQVEKVISHPNYDSKTKNNDIALMKLQKPLTFNDLVKPVCLPNPGMMLEPEQLCWISGWGATEEKGKTSEVLNAAKVLLIETQRCNSRYVYDNLITP
AMICAGFLQGNVDSCQGDAGGPLVTSKNNIWWLIGDTSWGSGCAKAYRPGVYGNVTVFTDWIYRQMRADSGGDYKDHDGDYKDHDIDYKDDDDK

Full length TMPRSS2 (S441A) marmoset XP_008984970.1 C-term 3XFLAG:
MALNSGSPPGVGPYYENHGYQPENPYSAQPTVAPNGYMVYPAQYYPSPLPQYAPRVPTHASNPVVSTQPKSPSGRLCTSKTKKALCITLALGTVLTGAALAAGLV
WKFMESKCSVSGIECGSSGTCVSPSNWCDGVSHCPSGEDENRCVRLYGPNFILQVYSSQRRSWHPVCQDDWNENYGRVACRDMGYKNSFYSSQGIVDDSGAT
SFMKLNLSAGNVDIYKKLYHSGTCSSKAVVSLRCVACGVTLNSSRQSRIVGGVTASEGAWPWQVSLHVQNVHVCGGSIITPEWIVTAAHCVEKPLNNPRHWTAFVG
ILSQSLMFYGSGHRVEKVISHPSYDSQTKNNDIALMKLQTPLTFSDAVKPVCLPNPGMNLDPEQPCWISGWGATEEKGKTSDMLNAAMVPLIDPQRCNSRYVYNNLI
TPAMICAGFLKGTVDSCQGDAGGPLVTLKNSVWWLIGDTSWGSGCAKAYRPGVYGNVTVFTDWIYQQMRADGSGDYKDHDGDYKDHDIDYKDDDDK

Full length TMPRSS2 (S441A) capuchin XP_032132713.1 C-term 3XFLAG:
MALNSGSPPGVGPYYENHGYQPENPYPAQPAVAPSVYMVYPAQHYPSPLPQYAPRVLTHASNPVVSTQPKSPSGRVCSSKTKKALCITLALGTVLAGAALAAGLV
WKFMESKCSVSEIECGSSGTCVSPSNWCDGVSHCPSGEDENRCVRLYGPNFILQVYSSQRRSWHPVCQDDWNENYGRAACRDMGYKNSFYSSQGIVDDSGAT
SFMKLNMSAGNVDIYKKLYHSDACFSKAVVSLRCIACGVTSNSSRQSRIVGGVRAPKGAWPWQVSLHVQNVHVCGGSIITPEWIVTAAHCVEKPLNNPRHWTAFVG
ILSQSLMFYGSGHRVEKVISHPSYDSQTKNNDIALMKLQAPLTFSDAVKPVCLPNPGMNLDPEQPCWISGWGATEEKGKTSDVLNAAMVPLIDPQRCNSRYVYNNLI
TPAMICAGFLKGTVDSCQGDAGGPLVTLKNSVWWLIGDTSWGSGCAKAYRPGVYGNVTVFTDWIYQQMRADGSGDYKDHDGDYKDHDIDYKDDDDK

Full length TMPRSS2 (S441A) flying fox XP_039713727.1 C-term 3XFLAG:
MALSSGSPPPSVGPYYENGGYQPDSLYPPRPTVVPTAFSVYPAPYYPPAVPQYTPRIPTSASTPASHVQPKSPSGTLCTSRTRKALCIACALGTVLVGAVVAAVLLW
KFMDNKCSVSGMECGSSGTCVTASQWCDGVLNCPSGEDENRCVRLYGPNFILQVYSSQRKSWHPVCQDDWSESYGRAACQDMGYRNSFYTSQGTADDSGAT
SFMKVNRSADDTDLYKKLYHSDVCSSKTVVSLRCIECGVNARMGVSRQSRIVGGTNAASGDWPWQVSLHVQGTHVCGGSIITPDWIVTAAHCVEEPLNNPRYWTA
FAGILRQSFMFYGSGYRVEKVISHPNYDSNTKNNDIALMKLQAPLTFTDKVKPVCLPNPGLMLEPTQPCWISGWGATYEKGKTSDVLNAAMVPLIEPWRCNNRYVY
NNLVTPAMICAGYLQGTVDSCQGDAGGPLVTLKSSIWWLIGDTSWGSGCAKAYRPGVYGNVTVFTDWIYRQMRANSGGDYKDHDGDYKDHDIDYKDDDDK

Full length TMPRSS2 (S441A) alpaca XP_031540159.1 C-term 3XFLAG:
MALNSGSPPGVGPYYENHGYQPESFYPLRPSAAPSAYMVYPAQYYPPAVPQYTPRVQTHTSTPVIRMQPKPPSETVCTSKTKKVLCVTLGLGAVLVGAVLAAVLL
WKFKESKCSASGMECGSSGTCISPSQWCDGILHCPGGEDENRCVRLYGPNFILQVYSPQRDSWHPVCQDDWSESFGRAMCQDLGYGNSFYSSQGVADDSGAT
SFMKLNISANNIDLYKKLYHSDVCSSKRVVSLRCIECGVSEKTSRQSRIVGGSSANLGDWPWQVSLHVQGIHVCGGSIITPEWIVTAAHCVERPLNNAKIWTAFAGILS
QSLMIYGNGYQIAKVISHPNYDSKTKNNDIALMKLQTPLTFNDRVKPVCLPNPGMMLETTQSCWISGWGATYEKGKTSDVLNAVMVHLIEPWKCNSKYVYNNLITSA
MICAGYLQGGVDSCQGDAGGPLVTLKNSVWWLLGDTSWGSGCAKAYRPGVYGNVTVFTDWIYRQMRANSGGDYKDHDGDYKDHDIDYKDDDDK

Full length TMPRSS2 (S441A) cow NP_001075054.1 C-term 3XFLAG:
MALNSGSSPGVGPYYENHGYQPESLYPQQPPTAPPAFEVYPAQYYPPVVPQYTPRVQTHASTPVIYRQPKPPSRTACTSKTKKALCITFTLVVLLAGAILAAVLLWK
YMEDECSGMECSSSGTCVSPSLWCDGILHCPSGEDENRCVRLYGPNFILQVYSAQRKSWHPVCQDDWSESYGRAACQDMGYRNSFYSSQGIADDSGATSFMKL
NISANDIDLYKKLYHSDVCSSKTVVSLRCIECGVSVKTSRQSRIVGGSNAYSGEWPWQVSLHVQGIHVCGGSIITPEWIVTAAHCVEEPLNNPKIWVAFAGILKQSYM
FYGSGYRVAKVISHPNYDSKTKNNDIALMKLQTPLTFNDKVKPVCLPNPGMMLEPTQSCWISGWGATYEKGKTSDDLNAAKVHLIEPRKCNSKYMYDNLITPAMICA
GYLRGTVDSCQGDAGGPLVTLKSSVWWLIGDTSWGSGCAKAYRPGVYGNVTVFTDWIYQQMRANSGGDYKDHDGDYKDHDIDYKDDDDK

Full length TMPRSS2 (S441A) mole rat XP_008825630.2 C-term 3XFLAG:
MALNSGSSPGIGPYYENHGYQPEHAYPQRPPVAPNVYQVYPAQDYPSSVPQYAPRVTTHTSTTSVIHRQPKSGEQCTSKTKKALCVTLTLGLVLAGAAVAAGLLWK
FLEDKCSESEMECSSSGKCISSSLWCDGISQCPNGEDENRCVRLYGPSFILQVFSSQRKSWHPVCQDDWNENYGRAACKDMGYKDSFYFSQGIPDNSGATSFMK
LNLSAANIDLYKKLYHSDTCSSRMVVSLRCIECGVRSVTRQSRIVGGSSASLTEWPWQVSLHVQGVHVCGGSIITPEWIVTAAHCVEEPLNSPRYWTAFAGISRQSL
MFYGKRHQVEKVISHPNYDSKTKNNDIALMKLQTPLSFNDVVKPVCLPNPGMMLDPDQECWISGWGATYEKGSTSDVLNAAMVPLIELSKCNNKYVYNNLITPAMIC
AGYIQGTIDSCQGDAGGPLVTWKNSIWWLIGDTSWGSGCAKAYRPGVYGNVTVFTDWIYGQMRANSGGDYKDHDGDYKDHDIDYKDDDDK

Full length TMPRSS2 (S441A) multimammate mouse (natal mouse) XP_031220699.1 C-term 3XFLAG:
MALNSGSPPGIGPYYENHGYQSEHIYPPRPPVAPNGYNLYPAQCYPSPVPQYAPRVTTQASTPAIHIQPKSSGTLCTSKSKKSLCVALALGTVLAGAAVVAGLLWKF
LPGDSKCFTSEMECGSSGTCISSSLWCDGVSHCPNGEDENRCVRLYGPSFTLQIYSSQRKAWYPVCQDDWSESYGRAACKDMGYKNNFYSSQGIPDHSGATSF
MKLNVSAGNIDLYKKLYHSDSCSSRLVVSLRCIECGVRSHSVRRQSRIVGGSNAAPGEWPWQVSLHVQGIHVCGGSIITPEWIVTAAHCVEEPLSSPRYWTAFAGIL
KQNLMFYGNRHQVEKVIPHPGYDSQTKNNDIALMKLQTPLAFNDVVKPVCLPNPGMMLDLNQECWISGWGATYEKGKTSDILNAAMVPLIEPSKCNSKYVYNNLVT
PAMICAGFLQGSVDSCQGDAGGPLVTSKNGIWWLIGDTSWGSGCAKAYRPGVYGNVTVFTDWIYQQMRANSGGDYKDHDGDYKDHDIDYKDDDDK

**TMPRSS2 ectodomain (S441A):**<br>MTRLTVLALLAGLLASSRASMGSKCSNSGIECDSSGTCINPSNWCDGVSHCPGGEDENRCVRLYGPNFILQVYSSQRKSWHPVCQDDWNENYGRAACRDMGY<br>KNNFYSSQGIVDDSGSTSFMKLNTSAGNVDIYKKLYHSDACSSKAVVSLRCIACGVNLNDDDDKIVGGESALPGAWPWQVSLHVQNVHVCGGSIITPEWIVTAAH<br>CVEKPLNNPWHWTAFAGILRQSFMFYGAGYQVEKVISHPNYDSKTKNNDIALMKLQKPLTFNDLVKPVCLPNPGMMLQPEQLCWISGWGATEEKGKTSEVLNAA<br>KVLLIETQRCNSRYVYDNLITPAMICAGFLQGNVDSCQGDAGGPLVTSKNNIWWLIGDTSWGSGCAKAYRPGVYGNVMVFTDWIYRQMRADGDDDDKSGHHHH<br>HHHH
**TMPRSS2 ectodomain (S441A + DS):**<br>MTRLTVLALLAGLLASSRASMGSKCSNSGIECDSSGTCINPSNWCDGVSHCPGGEDENRCVRLYGPNFILQVYSSQRKSWHPVCQDDWNENYGRAACRDMGY<br>KNNFYSSQGIVDDSGSTSFMKLNTSAGNVDIYKKLYHSDACSSKAVVSLRCIACGVNLNDDDDKIVGGESALPGAWPWQVSLHVQNVHVCGGSIITPEWIVTAAH<br>CVEKPLNNPWHWTAFAGILRQSFMFYGAGYQVEKVISHPNYDSKTKNNDIALMKLQKPLTFNDLVKPVCLPNPGMMLQPEQLCWISGWGATEEKGKTSEVLNAA<br>KVLLIETQRCNSRYVYDNLITPAMICAGFLQGNVDSCQGDAGGPLVCSKNNIWWLIGDTSWGSGCAKAYRPGVYGNVMVFTDWIYRQMRADGDDDDKSGHHHH<br>HHHH
**TMPRSS2 ectodomain (S441 + DS):**<br>MTRLTVLALLAGLLASSRASMGSKCSNSGIECDSSGTCINPSNWCDGVSHCPGGEDENRCVRLYGPNFILQVYSSQRKSWHPVCQDDWNENYGRAACRDMGY<br>KNNFYSSQGIVDDSGSTSFMKLNTSAGNVDIYKKLYHSDACSSKAVVSLRCIACGVNLNDDDDKIVGGESALPGAWPWQVSLHVQNVHVCGGSIITPEWIVTAAH<br>CVEKPLNNPWHWTAFAGILRQSFMFYGAGYQVEKVISHPNYDSKTKNNDIALMKLQKPLTFNDLVKPVCLPNPGMMLQPEQLCWISGWGATEEKGKTSEVLNAA<br>KVLLIETQRCNSRYVYDNLITPAMICAGFLQGNVDSCQGDSGGPLVCSKNNIWWLIGDTSWGSGCAKAYRPGVYGNVMVFTDWIYRQMRADGDDDDKSGHHHH<br>HHHH
**TMPRSS2 ectodomain (S441A + DS + SUMO):**<br>MTRLTVLALLAGLLASSRASMSDSEVNQEAKPEVKPEVKPETHINLKVSDGSSEIFFKIKKTTPLRRLMEAFAKRQGKEMDSLRFLYDGIRIQADQTPEDLDMEDND<br>IIEAHRENDDDDKTTGSKCSNSGIECDSSGTCINPSNWCDGVSHCPGGEDENRCVRLYGPNFILQVYSSQRKSWHPVCQDDWNENYGRAACRDMGYKNNFYS<br>SQGIVDDSGSTSFMKLNTSAGNVDIYKKLYHSDACSSKAVVSLRCIACGVNLNDDDDKIVGGESALPGAWPWQVSLHVQNVHVCGGSIITPEWIVTAAHCVEKPL<br>NNPWHWTAFAGILRQSFMFYGAGYQVEKVISHPNYDSKTKNNDIALMKLQKPLTFNDLVKPVCLPNPGMMLQPEQLCWISGWGATEEKGKTSEVLNAAKVLLIET<br>QRCNSRYVYDNLITPAMICAGFLQGNVDSCQGDAGGPLVCSKNNIWWLIGDTSWGSGCAKAYRPGVYGNVMVFTDWIYRQMRADGDDDDKSGHHHHHHHH
**TMPRSS2 ectodomain (S441 + DS + SUMO):**<br>MTRLTVLALLAGLLASSRASMSDSEVNQEAKPEVKPEVKPETHINLKVSDGSSEIFFKIKKTTPLRRLMEAFAKRQGKEMDSLRFLYDGIRIQADQTPEDLDMEDND<br>IIEAHRENDDDDKTTGSKCSNSGIECDSSGTCINPSNWCDGVSHCPGGEDENRCVRLYGPNFILQVYSSQRKSWHPVCQDDWNENYGRAACRDMGYKNNFYS<br>SQGIVDDSGSTSFMKLNTSAGNVDIYKKLYHSDACSSKAVVSLRCIACGVNLNDDDDKIVGGESALPGAWPWQVSLHVQNVHVCGGSIITPEWIVTAAHCVEKPL<br>NNPWHWTAFAGILRQSFMFYGAGYQVEKVISHPNYDSKTKNNDIALMKLQKPLTFNDLVKPVCLPNPGMMLQPEQLCWISGWGATEEKGKTSEVLNAAKVLLIET<br>QRCNSRYVYDNLITPAMICAGFLQGNVDSCQGDSGGPLVCSKNNIWWLIGDTSWGSGCAKAYRPGVYGNVMVFTDWIYRQMRADGDDDDKSGHHHHHHHH
**TMPRSS2 ectodomain (S441A + DS + SUMO + N249 glycan):**<br>MTRLTVLALLAGLLASSRASMSDSEVNQEAKPEVKPEVKPETHINLKVSDGSSEIFFKIKKTTPLRRLMEAFAKRQGKEMDSLRFLYDGIRIQADQTPEDLDMEDND<br>IIEAHRENDDDDKTTGSKCSNSGIECDSSGTCINPSNWCDGVSHCPGGEDENRCVRLYGPNFILQVYSSQRKSWHPVCQDDWNENYGRAACRDMGYKNNFYS<br>SQGIVDDSGSTSFMKLNTSAGNVDIYKKLYHSDACSSKAVVSLRCIACGVNLNSSDDDDKIVGGESALPGAWPWQVSLHVQNVHVCGGSIITPEWIVTAAHCVEK<br>PLNNPWHWTAFAGILRQSFMFYGAGYQVEKVISHPNYDSKTKNNDIALMKLQKPLTFNDLVKPVCLPNPGMMLQPEQLCWISGWGATEEKGKTSEVLNAAKVLLI<br>ETQRCNSRYVYDNLITPAMICAGFLQGNVDSCQGDAGGPLVCSKNNIWWLIGDTSWGSGCAKAYRPGVYGNVMVFTDWIYRQMRADGDDDDKSGHHHHHHH<br>H
**TMPRSS2 ectodomain (S441 + DS + SUMO + N249 glycan):**<br>MTRLTVLALLAGLLASSRASMSDSEVNQEAKPEVKPEVKPETHINLKVSDGSSEIFFKIKKTTPLRRLMEAFAKRQGKEMDSLRFLYDGIRIQADQTPEDLDMEDND<br>IIEAHRENDDDDKTTGSKCSNSGIECDSSGTCINPSNWCDGVSHCPGGEDENRCVRLYGPNFILQVYSSQRKSWHPVCQDDWNENYGRAACRDMGYKNNFYS<br>SQGIVDDSGSTSFMKLNTSAGNVDIYKKLYHSDACSSKAVVSLRCIACGVNLNSSDDDDKIVGGESALPGAWPWQVSLHVQNVHVCGGSIITPEWIVTAAHCVEK<br>PLNNPWHWTAFAGILRQSFMFYGAGYQVEKVISHPNYDSKTKNNDIALMKLQKPLTFNDLVKPVCLPNPGMMLQPEQLCWISGWGATEEKGKTSEVLNAAKVLLI<br>ETQRCNSRYVYDNLITPAMICAGFLQGNVDSCQGDSGGPLVCSKNNIWWLIGDTSWGSGCAKAYRPGVYGNVMVFTDWIYRQMRADGSGLNDIFEAQKIEWHE<br>QSGHHHHHHHH

Table S1. Amino acid sequences of designed TMPRSS2 constructs, related to Figure 1.
