# Supplementary material for: Human coronavirus HKU1 recognition of the TMPRSS2 host receptor
Source: Cell. Author manuscript; Available in PMC 2026 Jan 29. (PMC12854727; doi:10.1016/j.cell.2024.06.006)
Supplement: FigS5 — (A) Raw data used for Figure 3B; the upper left panel shows the color key used for the other panels. Data show measured fluorescence (RFU) over time with 3.4 nM TMPRSS2 (harboring the N-terminal SUMO fusion, the C379-T447C disulfide bond and the N249 glycan) incubated with 0.19 mM Boc-QAR-AMC at 22°C in the presence of various concentrations of HKU1 isolate N1 RBD and alanine interface mutant RBDs. (B) Raw data and calculations used for Figure 3C–D; the upper left panel shows the color key used for the other panels. Lower left panels show a summary of AMC standard curves used to calculate AMC release from the Boc-QAR-AMC peptide substrate with 215, 72, and 24 nM HKU1 RBD, accounting for the inner filter effect. The middle panels show measured fluorescence (RFU) over time with 5 nM TMPRSS2 and 215, 72, or 24 nM HKU1 RBD (wildtype) or 215 nM HKU1 R517A RBD, incubated with various concentrations of Boc-QAR-AMC at 22 °C. The right panel shows the change in calculated AMC concentration over time. [file NIHMS2094799-supplement-FigS5.pdf]

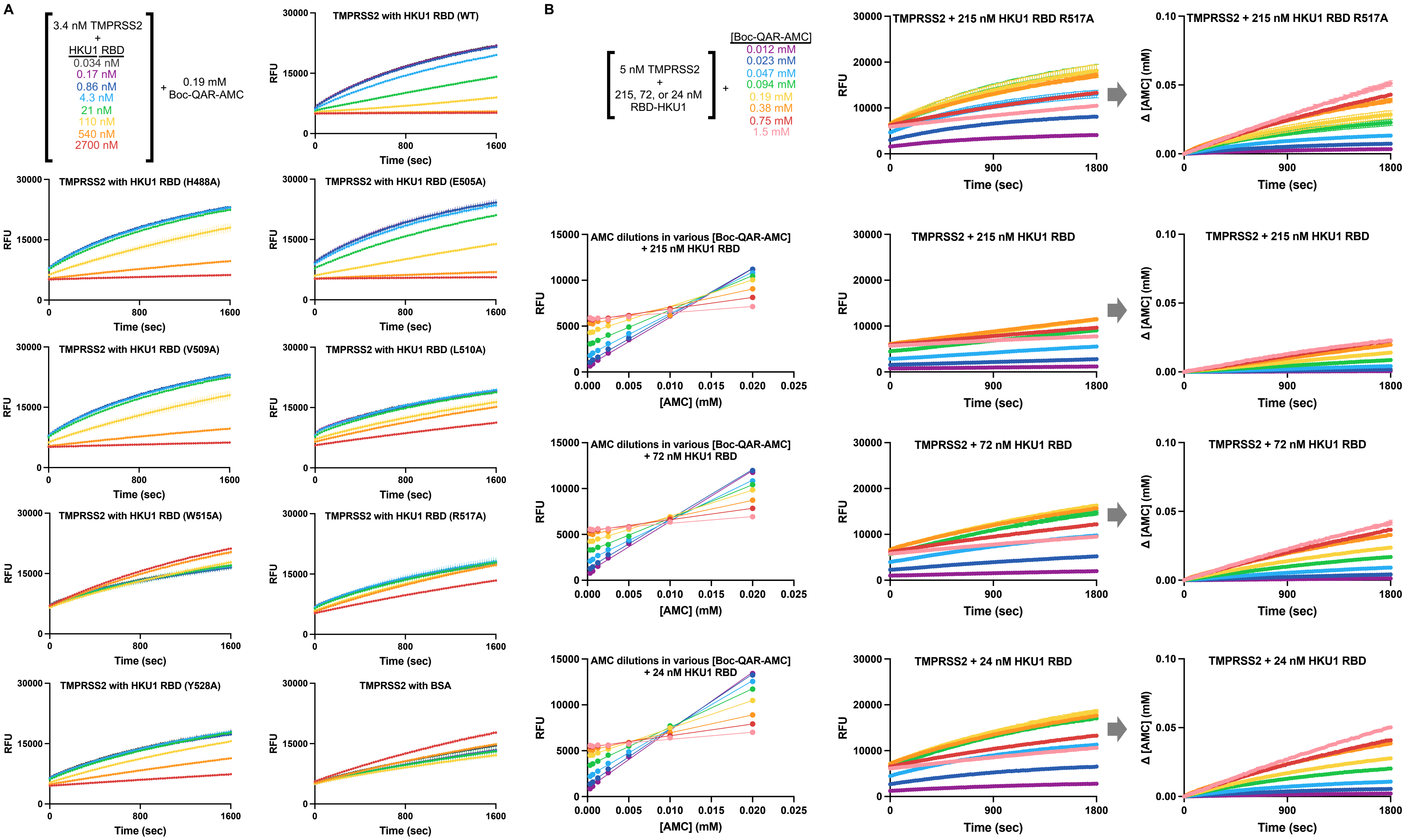

A
3.4 nM TMPRSS2
+
HKU1 RBD
0.034 nM
0.17 nM
0.86 nM
4.3 nM
21 nM
110 nM
540 nM
2700 nM
+ 0.19 mM Boc-QAR-AMC
TMPRSS2 with HKU1 RBD (WT)
TMPRSS2 with HKU1 RBD (H488A)
TMPRSS2 with HKU1 RBD (E505A)
TMPRSS2 with HKU1 RBD (V509A)
TMPRSS2 with HKU1 RBD (L510A)
TMPRSS2 with HKU1 RBD (W515A)
TMPRSS2 with HKU1 RBD (R517A)
TMPRSS2 with HKU1 RBD (Y528A)
TMPRSS2 with BSA
RFU
Time (sec)
B
5 nM TMPRSS2
+
215, 72, or 24 nM RBD-HKU1
+
[Boc-QAR-AMC]
0.012 mM
0.023 mM
0.047 mM
0.094 mM
0.19 mM
0.38 mM
0.75 mM
1.5 mM
TMPRSS2 + 215 nM HKU1 RBD R517A
AMC dilutions in various [Boc-QAR-AMC] + 215 nM HKU1 RBD
TMPRSS2 + 215 nM HKU1 RBD
AMC dilutions in various [Boc-QAR-AMC] + 72 nM HKU1 RBD
TMPRSS2 + 72 nM HKU1 RBD
AMC dilutions in various [Boc-QAR-AMC] + 24 nM HKU1 RBD
TMPRSS2 + 24 nM HKU1 RBD
[AMC] (mM)
Δ [AMC] (mM)
